# Supplementary material for: Ethnic minority women’s empowerment in agriculture in the central region of Viet Nam
Source: PLoS One. 2023 Aug 7;18(8):e0287115. doi: 10.1371/journal.pone.0287115 (PMC10406194; doi:10.1371/journal.pone.0287115)
Supplement: S1 File — (PDF) [file pone.0287115.s001.pdf]

PROVINCIAL CODE...............................

DISTRICT CODE.....................................

VILLAGE CODE.......................................

FAMILY CODE..

SEX CODE (1- male; 2 – female)..............

QUESTIONAIRE FOR EMPOWERMENT IN AGRICULTURE

(The survey is translated from the Vietnamese original)

Dear Mr/Ms!

Currently, Duy Tan University is conducting a research on empowering women in agriculture in ethnic minority areas in central Vietnam, thereby proposing policy solutions to improve women's rights this area.

We would very much like you to take the time to answer the questions in this questionnaire. Hope the exchange will bring you interesting feelings.

Your opinions are only for scientific research, and are not used for any other purpose.

Thank you very much for your cooperation!

PERSONAL INFORMATION

Could you please provide some information about yourself below?

1. Your date of birth? *(write in numbers)*...

2. What is the highest high school class you have (or are) taking? *(write in numbers)* ....

3. Number of children in your family? *(write in numbers)*...

4. What is the highest high school class that one of your children has (or is) taking? (write in numbers)...................

5. Could you please tell us the age of your first marriage?

1. Under 18 years old   2. From 18 to 22 years old
3. From 23 to 26 years old   4. From 27 to 30 years old
5. From 31 to 35 years old   6. Over 35 years old

6. What is your current marital status?

1. Monogamy   2. Divorced   3. Being a single dad   4. Being a single mom

7. What is the distance from your house to the nearest town (town, town, city)? (write in numbers)..........................

8. How many million VND is your family's monthly income? (write in numbers) ....

9. Ethnicity (inscribed in words)...

10. How often do you hear about gender equality messages?

1. Never heard   2. Glimpse of hearing   3. Can you hear?
4. Heard often   5. Heard it very often

11. Do you know anything about the Law on Gender Equality?

1. Didn't know 2. Knowing insignificant 3. Know a little 4. Know well 5. Know very well

12. What is your opinion on the view that “men and women must have the same conditions for development”?

1. Totally disagree 2. Disagree 3. Normal 4. Agree 5. Strongly agree

13. What is your opinion on the view that “men and women should have equal rights in all decisions in the family?

1. Totally disagree 2. Disagree 3. Normal 4. Agree 5. Strongly agree

MAIN CONTENTS

1. PRODUCTION

This section includes questions on two topics: (1) participation in agricultural activities and (2) decision-making regarding these activities.		How do you usually make decisions for the following activities?	How confident do you feel you can make decisions about activities?
**1**	**Growing food crops: (crops that mainly serve the needs of the family)**<br>*(Investigator switches to sentence 2 when the respondent chooses the option “No decision-making power”)*	(1A)<br>No decision-making power....................................1<br>Make decisions together with someone other than a family member ……...2<br>Make decisions together with a family member...3<br>Husband and wife make decisions together…........4<br>Make own decisions ………………...............….5	(1B)<br>Very unconfident ……..**1**<br>Not confident ……..…...**2**<br>Normal ....………..…**3**<br>Pretty confident ……..…**4**<br>Very confident ……….**5**

**2**	**Commercial crops: (crops for which the post-harvest product can be traded)**<br>*(Investigator switches to question 3 when the respondent chooses the option "No decision-making power")*	(2A)<br>No decision-making power....................................1<br>Make decisions together with someone other than a family member ……...2<br>Make decisions together with a family member...3<br>Husband and wife make decisions together…........4<br>Make own decisions ……........………….......…..5	(2B)<br>Very unconfident ……..**1**<br>Not confident ………...**2**<br>Normal ....……...…**3**<br>Pretty confident …...…**4**<br>Very confident ……….**5**
**3**	**Livestock activities**<br>*(Investigator switches to question 4 when the respondent chooses the option "No decision-making power")*	(3A)<br>No decision-making power....................................1<br>Make decisions together with someone other than a family member ……...2<br>Make decisions together with a family member...3<br>Husband and wife make decisions together…........4<br>Make own decisions ……........………….......…..5	(3B)<br>Very unconfident ……..**1**<br>Not confident ………...**2**<br>Normal ....……...…**3**<br>Pretty confident …...…**4**<br>Very confident ……….5

**4**	**Paid Employment: (paid in cash or in kind from activities related to agriculture or activities other than agriculture)**<br>*(Investigator switches to question 5 when the respondent chooses the option "No decision-making power")*	(4A)<br>No decision-making power.....................................1<br>Make decisions together with someone other than a family member ……...2<br>Make decisions together with a family member...3<br>Husband and wife make decisions together…........4<br>Make own decisions ……........………….......…..5	(4B)<br>Very unconfident ……..**1**<br>Not confident ………...**2**<br>Normal ....……...…**3**<br>Pretty confident ….....…**4**<br>Very confident ……….5
**5**	**Large household spending activities (such as spending on vehicles or land, etc.)**<br>*(Investigator switches to question 6 when the respondent chooses the option "No decision-making power")*	(5A)<br>No decision-making power.....................................1<br>Make decisions together with someone other than a family member ……...2<br>Make decisions together with a family member...3<br>Husband and wife make decisions together…........4<br>Make own decisions ……........………….......…..5	(5B)<br>Very unconfident ……..**1**<br>Not confident ………...**2**<br>Normal ....……...…**3**<br>Pretty confident ….....…**4**<br>Very confident ……….5
		(6A)<br>No decision-making power.....................................1	(6B)<br>Very unconfident ……..**1**<br>Not confident ………...**2**

**6**	**Ordinary household spending activities (such as spending on food, daily necessities, etc.)**	Make decisions together with someone other than a family member ……..2<br><br>Make decisions together with a family member...3<br><br>Husband and wife make decisions together….......4<br><br>Make own decisions ……........…………........…..5	Normal ....……...…**3**<br><br>Pretty confident …....…**4**<br><br>Very confident ……….5

2. RESOURCES

This section covers questions regarding access to and ownership of certain types of assets (in the form of capital) to generate income.		What rights do you have in owning the following properties?
**7**	**Agricultural land (plot/piece)**	No ownership ....1<br>Shared ownership with another person who is not a family member...................**2**<br>Shared with a family member ....3<br>Husband and wife jointly own..4<br>Own it alone 5
**8**	**Large livestock (cattle, poultry, etc.)**	No ownership ....1<br>Shared ownership with another person who is not a family member...................**2**<br>Shared with a family member ....3<br>Husband and wife jointly own..4<br>Own it alone 5
**9**	**Medium livestock (goats, pigs, sheep, etc.)**	No ownership ....1<br>Shared ownership with another person who is not a family member...................**2**<br>Shared with a family member ....3<br>Husband and wife jointly own..4<br>Own it alone 5

**10**	**Small animals (chickens, ducks, pigeons, etc.)**	No ownership ...1<br>Shared ownership with another person who is not a family member...**2**<br>Shared with a family member ...3<br>Husband and wife jointly own...4<br>Own it alone ...5
**11**	**Fish ponds and fishing gear**	No ownership ...1<br>Shared ownership with another person who is not a family member...**2**<br>Shared with a family member ...3<br>Husband and wife jointly own...4<br>Own it alone ...5
**12**	**Non-motorized agricultural implements (hand tools, or manual plows or harrows drawn by animals)**	No ownership ...1<br>Shared ownership with another person who is not a family member...**2**<br>Shared with a family member ...3<br>Husband and wife jointly own...4<br>Own it alone ...5
**13**	**Agricultural machinery (plows, tractors, cultivators, water pumps, etc.)**	No ownership ...1<br>Shared ownership with another person who is not a family member...**2**<br>Shared with a family member ...3

		Husband and wife jointly own....4<br>Own it alone 5
**14**	**Non-agricultural equipment (sewing machines, dispensing equipment, dryers, etc.)**	No ownership ....1<br>Shared ownership with another person who is not a family member....**2**<br>Shared with a family member ....3<br>Husband and wife jointly own....4<br>Own it alone 5
**15**	**Housing or other constructions**	No ownership ....1<br>Shared ownership with another person who is not a family member....**2**<br>Shared with a family member ....3<br>Husband and wife jointly own....4<br>Own it alone 5
**16**	**Large durable goods (refrigerator, TV, sofa, etc.)**	No ownership ....1<br>Shared ownership with another person who is not a family member....**2**<br>Shared with a family member ....3<br>Husband and wife jointly own....4<br>Own it alone 5
**17**		No ownership 1

	**Small durable appliances (transistors, microwaves, etc.)**	Shared ownership with another person who is not a family member................…**2**<br>Shared with a family member ....3<br>Husband and wife jointly own...………......4<br>Own it alone ………..……...............……….............5
**18**	**Điện thoại**	No ownership ....1<br>Shared ownership with another person who is not a family member................…**2**<br>Shared with a family member ....3<br>Husband and wife jointly own...………......4<br>Own it alone ………..……...............……….............5
**19**	**Non-agricultural land (residential land or business and service land)**	No ownership ....1<br>Shared ownership with another person who is not a family member................…**2**<br>Shared with a family member ....3<br>Husband and wife jointly own...………......4<br>Own it alone ………..……...............……….............5
**20**	**Means of transport (bicycles, motorbikes, cars, etc.)**	No ownership ....1<br>Shared ownership with another person who is not a family member................…**2**<br>Shared with a family member ....3<br>Husband and wife jointly own...………......4

		Own it alone ………..….................……….............5

3. CREDIT

This section includes questions related to financial performance (borrowing money) within the past 12 months.		If there is a need to borrow (including cash or in kind) can one of the family members get a loan from the following sources?	If it is possible to borrow, with whom are loan decisions usually made?
		**21A**	**21B**
**21**	**From non-governmental organizations (NGOs)**	Can't borrow at all.............................1<br>Not sure if can borrow......................2<br>Can borrow a little............................3<br>Borrowed..4<br>Sure can borrow 5	No decision-making power...1<br>Make decisions together with someone other than a family member ……..2<br>Make decisions together with a family member.... ....3<br>Husband and wife make decisions together…..................4<br>Make own decisions ………………...........................….5
**22**	**From official credit institutions (banks or local credit unions)**	**22A**<br>Can't borrow at all.............................1<br>Not sure if can borrow......................2<br>Can borrow a little............................3<br>Borrowed..4<br>Sure can borrow 5	**22B**<br>No decision-making power...1<br>Make decisions together with someone other than a family member ……..2<br>Make decisions together with a family member.... ....3<br>Husband and wife make decisions together…..................4

			Make own decisions ………………..........................…..55
**23**	**Friends or relatives, relatives**	Can't borrow at all.............................1<br>Not sure if can borrow......................2<br>Can borrow a little............................3<br>Borrowed..4<br>Sure can borrow 5	No decision-making power...1<br>Make decisions together with someone other than a family member ……...2<br>Make decisions together with a family member.... ....3<br>Husband and wife make decisions together…..................4<br>Make own decisions ………………..........................…..5

4. SOCIAL PARTICIPATION

This section includes questions about participating in groups and organizations in the community. These groups include formal, informal or groups related to customs and practices.		How often do you join the following groups/organizations?	Are you an active member?
	TYPE OF GROUP / ORGANIZATION PARTICIPATION		
**24**	Agricultural, livestock and aquaculture organizations (including trading organizations)	24A<br>Absolutely not..................1<br>Sometimes........................2<br>Medium............................3<br>Frequent...........................4<br>Very often........................5	24B<br>Very not positive................1<br>Quite positive.....................2<br>Normal...............................3<br>Positive..............................4<br>Very positive......................5
25	Forestry groups and organizations	25A<br>Absolutely not..................1<br>Sometimes........................2<br>Medium............................3<br>Frequent...........................4<br>Very often........................5	25B<br>Very not positive................1<br>Quite positive.....................2<br>Normal...............................3<br>Positive..............................4<br>Very positive......................5

26	Credit unions or financial groups (including credit unions and surnames)	26A<br>Absolutely not..................1<br>Sometimes........................2<br>Medium............................3<br>Frequent...........................4<br>Very often........................5	26B<br>Very not positive................1<br>Quite positive.....................2<br>Normal...............................3<br>Positive..............................4<br>Very positive......................5
27	Insurance or community support groups, organizations	27A<br>Absolutely not..................1<br>Sometimes........................2<br>Medium............................3<br>Frequent...........................4<br>Very often........................5	27B<br>Very not positive................1<br>Quite positive.....................2<br>Normal...............................3<br>Positive..............................4<br>Very positive......................5
28	Groups, trade associations, business	28A<br>Absolutely not..................1<br>Sometimes........................2<br>Medium............................3<br>Frequent...........................4<br>Very often........................5	28B<br>Very not positive................1<br>Quite positive.....................2<br>Normal...............................3<br>Positive..............................4<br>Very positive......................5

29	Community support groups or charity groups	Absolutely not..................1<br>Sometimes........................2<br>Medium............................3<br>Frequent...........................4<br>Very often........................55	Very not positive................1<br>Quite positive.....................2<br>Normal...............................3<br>Positive..............................4<br>Very positive......................5
30	Religious groups and organizations	Absolutely not..................1<br>Sometimes........................2<br>Medium............................3<br>Frequent...........................4<br>Very often........................5	Very not positive................1<br>Quite positive.....................2<br>Normal...............................3<br>Positive..............................4<br>Very positive......................5

5. USE OF TIME

In this section, individuals' activity time is calculated within 24 hours (activities are counted from 4 am to 4 am the next day). The entire operation time is divided into small intervals of 1 hour each. The enumerator will represent the respondent's results by hyphenating the activity periods.

Sleep and rest	4	5	6	7	8	9	10	11	12	13	14	15	16	17	18	19	20	21	22	23	0	1	2	3	4
Eating																									
Take care of yourself																									
Study																									

Hire																									
Self-trade																									
Cultivation/livestock																									
Shopping and wellness activities																									
Cooking																									
Housework																									
Taking care of children																									
Travel and meet friends																									
Watch TV, listen to radio, listen to music, read newspapers																									
Do exercise																									
Social activities and hobbies																									
Religious activities																									
Other activities																									
