# Supplementary material for: Ethnic minority women’s empowerment in agriculture in the central region of Viet Nam
Source: PLoS One. 2023 Aug 7;18(8):e0287115. doi: 10.1371/journal.pone.0287115 (PMC10406194; doi:10.1371/journal.pone.0287115)
Supplement: Attachment — Submitted filename: Reviewer comments_PONE-D-21-33301R2.pdf [file pone.0287115.s005.pdf]

<u>Reviewer comments</u>:

Ethnic minority women’s empowerment in agriculture in the central region of Viet Nam

Research plays an important role in assessing differences in the empowerment of men and women in the ownership and use of agricultural land. Research to fill in gaps in land use research and policy formulation in Vietnam. The study still has a few points that need to be added and improved as follows:

1. The title does not fully reflect the content of the study. The title should reflect gender differences in relation to agricultural land tenure and decisions about agricultural activities. That's because in all sections, including results, discussions and conclusions, there are clearly presented differences between men and women related to these activities. I therefore suggest that the abstract part also be rewritten accordingly.

2. Introduction should be provided with information about Vietnam's social context on women's and men's empowerment in terms of property ownership, power between men and women in social life and employment, especially in terms of agricultural land use. In addition, policies on agricultural land use rights are provided in more detail so that readers can better understand this field in Vietnam. From there, give the significance of the research when practiced in Vietnam.

3. Research data: Authors need to supplement information on economic, social, cultural characteristics, agricultural land use rates, occupational patterns in the localities to collect data to help readers understand why research to decide on the selection of information collection in these localities.

4. Data analysis: I think the results of the logistic regression model are not strong enough. Because factors from the husband's side can play very important role related to women' empowerment in the agricultural sector, such as husband's education, age gap between husband and wife, and husband's occupation. In Vietnamese culture, women are required to be subordient for 3 men in their life, including father's subordient when unmarried, husband's subordient when married and son's subordient when husband

died. Therefore, the regression model is flawed when it does not take into account the husband's characteristics in women's empowerment.

5. **Policy recommendations**: As I mentioned in the analysis of the results, because the research is lacking in taking into account the husband's characteristics in women's empowerment. Therefore, suggesting policies of this study may be flawed if only focusing on policies for women, while Vietnamese women are still heavily influenced by their husbands and in-laws.
