# Supplementary material for: Ethnic minority women’s empowerment in agriculture in the central region of Viet Nam
Source: PLoS One. 2023 Aug 7;18(8):e0287115. doi: 10.1371/journal.pone.0287115 (PMC10406194; doi:10.1371/journal.pone.0287115)
Supplement: Attachment — Submitted filename: renamed_2966d.doc [file pone.0287115.s002.doc]

Comments

**Abstract -**

"The results showed that up to 70% of 36 women lose their empowerment in the final empowerment index".

My comments:

More appropriate to state that 70% of the women had low empowerment. Throughout the paper (in the introduction, results and discussion sections), the authors mentioned women and men lose their empowerment- can we assume that these women had some powers but lost these later on? I suggest the authors rewrite these statements.

The results of the logistic regression can be presented more succinctly as what factors are significant in predicting women empowerment and what factors are not. Why single out age at marriage and children's education as the only two novel factors? Children's education is a meaningful only if they have grown-up children. As more than half of the respondents are below mid-30s, their children are too young to go to secondary school or institutions of higher education.

**Introduction**

"Line 51. Eighty-five percent of the global workforce is concentrated in the agricultural sector, of which females account for nearly a half (5)".

My comment: Wrong citation of the facts. Instead of global workforce, it should be the workforce in Ethiopia, as stated in article (5) in the reference list. The authors should check all the references to ensure that they have cited correctly.

"Line 53.. women’s empowerment in agriculture, especially those of ethnic minorities, still face gender inequality"

My suggestion : It should be women in agriculture instead of women's empowerment facing gender inequality.

"Lines 64-65. These studies showed that women often lost their right to property ownership, including land, capital, and durable goods [14]"

My comment: The loss of rights was stated in several places in the article- I do not agree with the use of the term losing their right as this assumes that they had the rights and then lost it. Why not "most women do not have the rights to...." or express it more aptly.

"Line 71. ...that educational attainment and household income were the most constant variables [17–24"

My comment: Inappropriate use of the term constant variables- consider using the terms "most commonly cited variables"

"Lines 73-75 some other exogenous factors such as the quality of family relationships, housing area, non-farm employment, and the distance to the nearest urban areas with a lesser extend"

My suggestion- rewrite as and to a less extend, some other exogenous factors such as.....

**Materials and Methods**

Line 106-107. A total of 150 households were selected in each of the three communes with the highest percentage of ethnic minorities of ......".

My comment: It will be good to mention the total population (number of households" in these communities, and justification why a sample size of 150 households is considered adequate for the study. One must note that interviewing two persons from the same household will result in high rate of homogeneity and design effect with higher margin of error.

"Line 118. As a result, a total of 400 questionnaires were collected, and only 300 satisfactory questionnaires were used for further analyses"

My comment -this may introduce some biases.

**Statistical analysis**

My comment: Any reason for not using ordinal regression?

**Characteristics of research samples**

"Lines 161-163. Especially, the rate of child marriage was more than three times higher in females (23.9%) than 162 in males (7.6%). The average age of respondents was 36.1 years old, in which the average age of 163 females (37.95 yo) was higher than males (34.55 yo) (*p* = 0.00)"

My comments :

1. Authors read the table wrongly. Females (34.6) are younger than males (38 years). Use one decimal place, instead of 2.

2, The average number of years of children's education is 5.4 years, indicating that most are in primary schools due to their young age. It will not provide a meaningful comparison between younger and older respondents, as the latter would have older children, and hence more likely to have more years of schooling.

**Participation in agricultural production activities**

Figures 1 and 2

My comments- Need to explain these more clearly. One can also present the different views from males and females on women's rights and empowerment. Do these figures represent the assessment by both genders, or males on males, and females on females?

"Lines 201-201 The overall average value of participation in agricultural activities, which was found to be 0.62 in males and 0.48 in females, had further confirmed the gender differences in empowerment (*p* = 0.00). At the cut-off of 0.6, the loss of women's rights was significantly higher (78.1%) as compared to men (41.4%). At the cut-off of 0.8, women lose their rights completely (100%) while that proportion in men was 89% (Fig 2A).

My comment: It will be more appropriate to state that "with a cut-off of 0.6, only 21.9% of the women achieved this threshold, compared to 58.4% of the males. None of the women had achieved a threshold of 0.8 compared to 11% of the males.

The same suggestion applies to other parts of the article.

**Own resources - should add access to loan in the sub-title**

Need to make reference to tables 3 and 4 in the text.

The description is rather confusing, and it is very hard to relate the numbers mentioned in the text and the tables. Need to write more clearly. Show statistical significance.

For instance, "The overall average value of the ownership of the resources had also shown the gender difference, with values of 6.1/10 for men and 4.4/10 for women (*p* = 0.00) - I cannot find these figures in the tables.

**Control over the use of income**

Table 5 under this sub-section shows decision-making on income generation, not on the use of income -wrong label?

**Evaluation of the empowerment’s affected factors by the logistic regression**  **model**

Ordinal regression may be a good alternative. Explain why this was not used.

Authors may present a cross-tab to show the average score or percent with a score of 0.6 and over by the independent variables, and conduct statistical test. The age factor may be significant at the bivariate level, but its effect is explained away by number of children. The effect of age at first marriage may be attenuated after adjusting for other variables in the model.

Show crude odds and adjusted odds ratios

Categorical independent variables (recoded from the ratio scale variables) - may show the effects of these variables more clearly (the significant differences across the various categories).

**Figures 2A - 2F - Label the figures.**

**Discussion**

"Line 346 loss of credit decisions...."

My comment - This again assume that the women had the autonomy to make credit decision or access credit, but lost it"

" Line 355-356 ....number of previous studies in Vietnam and other developing countries, however, claimed that women were whom to hold money and to decide how to use them [10,11"

My comments: Most studies found that women and men make joint decision, not sole decision on household spending and on buying propoerties.

"Line 372-375 As mentioned above, due to men’s chauvinism, few men volunteered to do housework with their wives. They often shun the housework and considered it a women's job. This is also the main reason why women do not have much rest and leisure time to recreate labor power".

My comment: More like gender role stereotyping, not so much male chauvinism.

"Lines 382-383. ...most of the empowerment studies [18,20–22,24,34], the age at first marriage, the highest education of children detected in this study were novel factors..."

My comments: Women's age at marriage is closely related to educational level.

**Conclusion and Policy Recommendations**

"Lines 396-397. Firstly, in the present study, we found that among the five assessed factors, the rate of women's disempowerment was significantly higher than that of men."

My suggestion: Women in rural agricultural setting were played a lesser role than the men, across socio-demographic variables.
