# Supplementary material for: Ethnic minority women’s empowerment in agriculture in the central region of Viet Nam
Source: PLoS One. 2023 Aug 7;18(8):e0287115. doi: 10.1371/journal.pone.0287115 (PMC10406194; doi:10.1371/journal.pone.0287115)
Supplement: Attachment — Submitted filename: Response to Reviewers (1).doc [file pone.0287115.s003.doc]

19 October, 2022

To

Editor-in-Chief,

Plos One.

Dear Editor,

With respect to your email dated Jul 8, 2022, enclosing the reviewers’ comments on the manuscript entitled “Ethnic minority women’s empowerment in agriculture in the central region of Vietnam and its associated factors” (Ref. No.: PONE-D-21-33301),

We would like to thank the Editor and reviewers for the insightful review and valuable comments on our manuscript. In the revised version, we hope that we have satisfactorily addressed the comments raised by the Editor and reviewers.

We hope you now will find the manuscript suitable for publication and look forward to hearing from you.

Your sincerely,

Thanh Tung Le

On behalf of all authors

**Editorial board’s comments**

***1.*** *Your manuscript has been assessed by two expert reviewers, whose comments are appended below. The reviewers have highlighted concerns about several aspects of the methodology and discussion, among other issues. Please ensure you respond to each point carefully in your response to reviewers document, and modify your manuscript accordingly.*

***Response:*** Thank you for your comment. we have provided a point-to-point answer to all remarks of reviewers. All the changes have been highlighted in the manuscript in red and a highlighted version with line numbers was also provided. We hope the manuscript after careful revisions meet reviewers high standards

***2.*** *In addition, the manuscript requires detailed copyediting to address errors in grammar, spelling and punctuation. We strongly recommend you consult a professional scientific editing service to improve the clarity and flow of your writing.*

***Response:*** Thank you for your suggestion. All spelling errors have now been corrected in the revised manuscript. The manuscript has also been proofreaded by two researchers with extensive experience in completing manuscripts for publication. I truly hope that the manuscript is now meet the standard of PLOS ONE.

***3.*** *Please change "female” or "male" to "woman” or "man" as appropriate, when used as a noun (see for instance* [*https://apastyle.apa.org/style-grammar-guidelines/bias-free-language/gender*](https://apastyle.apa.org/style-grammar-guidelines/bias-free-language/gender)*).*

***Response:*** Thank you for your great suggestion. We changed the phrase "female" or "male" to "woman" or "man" in all parts of the manuscript. Changes have been in lines: 51, 148, 149, 153, 156, 157, 159, 160, 162, 165, 175, 176, 181, 182, 184, 197, 207, 213, 214, 215, 228, 231, 232, 248, 250.

***4.*** *Thank you for stating the following in your Competing Interests section: "NO authors have competing interests"*

*Please complete your Competing Interests on the online submission form to state any Competing Interests. If you have no competing interests, please state "The authors have declared that no competing interests exist.", as detailed online in our guide for authors at* [*http://journals.plos.org/plosone/s/submit-now*](http://journals.plos.org/plosone/s/submit-now)

***Response:*** Thank you for your important suggestion*.* The all authors in this manuscript has declared that no competing interests exist. We have also completed all Plot 1 copyright request on this manuscript.

***5.*** *In your Data Availability statement, you have not specified where the minimal data set underlying the results described in your manuscript can be found. PLOS defines a study's minimal data set as the underlying data used to reach the conclusions drawn in the manuscript and any additional data required to replicate the reported study findings in their entirety. All PLOS journals require that the minimal data set be made fully available. For more information about our data policy, please see* [*http://journals.plos.org/plosone/s/data-availability*](http://journals.plos.org/plosone/s/data-availability)*.*

***Response:*** Thank you for the valuable comment. We believe that, data is always necessary to be clear so, we provided original data as evidence of all our research result. We have provided the data of the manuscript to datadryad's system. Our data can be found on the link or DOI code following:

<https://datadryad.org/stash/share/1ZDAdufsftSUucHV8x3yjpWeGcQ_Unee0eKD2sVVOEMd>

DOI: 10.5061 / dryad.dbrv15f4q

**Thank you very much for all your valuable comments and suggestions!**

19 October, 2022

To

Reviewer #1

Dear Reviewer,

We would like to thank the reviewer for the insightful review and valuable comments on our manuscript. In the revised version, we hope that we have satisfactorily addressed the comments raised by the reviewer.

Here below, we provide a point-to-point answer to all remarks. All the changes have been highlighted in the manuscript in red and a clean version with line numbers was also provided.

We would like to thank the reviewer again for taking the time to review our manuscript. We hope the manuscript after careful revisions meet your high standards. We are happy to respond to any further questions and comments you may have.

Your sincerely,

Thanh Tung Le

On behalf of all authors

**Reviewer #1**

***1.*** *“The results showed that up to 70% of 36 women lose their empowerment in the final empowerment index”.*

*More appropriate to state that 70% of the women had low empowerment. Throughout the paper (in the introduction, results and discussion sections), the authors mentioned women and men lose their empowerment- can we assume that these women had some powers but lost these later on? I suggest the authors rewrite these statements?*

***Response:*** Thank you for the suggestion. The term “lose empowerment” has now been changed as per your suggestion throughout the revised manuscript. Changes have been highlighted in red as the following.

***Revises text:***

- “The results showed that up to 70% of women did not reach the threshold of empowerment” (Lines: 37 – 38).

- These studies showed that women often generally have fewer rights than men to property ownership, including land, capital, and durable goods (Lines: 63 – 65).

- With a cut-off of 0.6, only 21.9% of the women achieved this threshold, compared to 58.4% of the men. None of the women had achieved a threshold of 0.8 compared to 11% of the men (Fig 2A) (Lines: 194 - 195).

- With a cut-off of 0.6, only 5.5 % of the women achieved this threshold, compared to 60.6% of the men. 5.5% of the women had achieved a threshold of 0.8 compared to 66.5 % of the men (p = 0.00) (Fig 2C). (Lines: 245 - 247).

- There was no statistically significant difference between men and women in the social participation. With a cut-off of 0.6, 21.9 % of the women achieved this threshold, compared to 27.9 % of the men (p > 0.05). Similarly, at the cut - off of 0.8, there was no also statistically difference significant. (Fig 2D) (Lines: 259 - 262).

- With a cut-off of 0.6, only 29.4 % of the women achieved this threshold, compared to 41.2 % of the men. 1.4 % of the women had achieved a threshold of 0.8 compared to 23.7 % of the men (p = 0.00) (Fig 2E) (Lines: 269 – 271).

- The general assessment of the above 5 indicators revealed a big difference in the empowerment between men and women (p = 0.00). With a cut-off of 0.6, only 30.3 % of the women achieved this threshold, compared to 84.8 % of the men. Only 19.4 % of the women had achieved a threshold of 0.8 compared to 53.8 % of the men (p = 0.00) (Fig 2F) (Lines: 273 – 276).

- Because the percentage of both men and women not reaching the empowerment threshold of 0.8 is very high (Lines: 286 – 287).

- In addition, our results also indicated that most of the women in the rural area have less rights than men of property ownership, especially for valuable properties such as farm equipment and non-agricultural land (Lines: 322 – 324).

- Firstly, in the present study, we found that in all five assessed factors Women in rural agricultural setting were played a lesser role than the men. Resource ownership, spend time, and household financial control were are typical elements. (Lines 379 – 381).

- Secondly, 70% of women in this study didn't reach the empowered threshold in agriculture signified a large gender gap in these specific geographic areas, and suggested an urgent need for specific and timely policy interventions to increase women's empowerment in these regions (Lines: 382 – 384).

***2.*** *The results of the logistic regression can be presented more succinctly as what factors are significant in predicting women empowerment and what factors are not.*

***Response:*** Thank you for your comment. The results of the logistic regression has been represented as per your suggestion**.**

***Revises text:*** The regression analyses using the binary logistic model revealed the age at first marriage, the level of children's education, education level, distance to the nearest urban area, and the number of children were related on the probability of empowerment. Age, income, and the level of gender awareness did not show any related (Lines: 38 - 42).

***3****. Why single out age at marriage and children's education as the only two novel factors?*

***Response:*** To the best of our knowledge, not like the other factors, these two factors have never been identified by any other previous studies.

***4.*** *Children's education is a meaningful only if they have grown-up children. As more than half of the respondents are below mid-30s, their children are too young to go to secondary school or institutions of higher education.*

***Response:*** Thank you for your comment. During the pilot survey (April, 2018), we found that children of ethnic minority households had years of schooling that did not match their age. This is because children in this area enter the first grade much later than those of other areas. For example, there are many children of the interviewed households who are already 14 or 15 years old but are only at 5^th^ grade. Moreover, the percentage of students attending secondary school in this area is low. In many households, children dropped out of school halfway, or only finish first grade, or even don't go to school at all. The main reasons given area: poverty, long distance from school, language barrier. As a result, for this area, even just knowing how to read and write has already made a big difference. This finding also coincides with several other recent studies on ethnic minorities in Vietnam.

We have tested this variable and found that it really makes sense in our regression model. This could be explained by the result of the interaction between children and parents. We will continue to test this variable in the future studies on other ethnic minorities in Vietnam and hopefully, it will be a novel and meaningful variable in policy recommendations.

***5.*** *“Line 51. Eighty-five percent of the global workforce is concentrated in the agricultural sector, of which females account for nearly a half (5)”.*

*My comment: Wrong citation of the facts. Instead of global workforce, it should be the workforce in Ethiopia, as stated in article (5) in the reference list. The authors should check all the references to ensure that they have cited correctly.*

***Response:*** Thank you for pointing this out. The text has been edited accordingly and changes have been highlighted in red. We have also gone through the reference list and confirm that all the references have been cited correctly.

***Revised text:*** A study conducted recently in Ethiopia in Africa showed that eighty-five percent of the population is concentrated in the agricultural sector, of which females are accounted for nearly a half. (Lines: 50 – 52).

***6.*** *"Line 53. women’s empowerment in agriculture, especially those of ethnic minorities, still face gender inequality"*

*My suggestion: It should be women in agriculture instead of women's empowerment facing gender inequality.*

***Response:*** Thank you for pointing this out. This sentence has now been revised as per your suggestion.

***Revised text:*** However, studies indicated that women in agriculture still face gender inequality, including gender inequality in: property ownership, right of financial access, social social participation, and time usage [2,6,7]. (Lines: 52 – 54).

***7.*** *"Lines 64-65. These studies showed that women often lost their right to property ownership, including land, capital, and durable goods [14]".*

*My comment: The loss of rights was stated in several places in the article - I do not agree with the use of the term losing their right as this assumes that they had the rights and then lost it. Why not "most women do not have the rights to...." or express it more aptly.*

***Response:*** the sentence has now been edited in the revised manuscript as per your suggestion.

***Revised text:*** These studies showed that women often have fewer rights than men to property ownership, including land, capital, and durable goods. (Lines: 63 – 65).

***8.*** *"Line 71. ...that educational attainment and household income were the most constant variables [17–24"].*

*My comment: Inappropriate use of the term constant variables- consider using the terms "most commonly cited variables".*

***Response:*** Thank you for your suggestion. The sentence has now been revised accordingly.

***Revised text:*** A number of the previous studies using a Logit regression model to assess the probability of empowerment-related factors revealed that educational attainment and household income were the most commonly cited variables. (Lines: 68 – 70).

***9.*** *"Lines 73-75 some other exogenous factors such as the quality of family relationships, housing area, non-farm employment, and the distance to the nearest urban areas with a lesser extend"*

*My suggestion- rewrite as and to a less extend, some other exogenous factors such as.....*

***Response:*** This sentence has now been rewrite as per your suggestion.

***Revised text:*** and some other exogenous factors such the quality of family relationships, housing area, non-farm employment, and the distance to the nearest urban areas with a lesser extent. (Lines: 72 – 74).

***10.*** *Line 106-107. A total of 150 households were selected in each of the three communes with the highest percentage of ethnic minorities of ......".*

*My comment: It will be good to mention the total population (number of households" in these communities, and justification why a sample size of 150 households is considered adequate for the study. One must note that interviewing two persons from the same household will result in high rate of homogeneity and design effect with higher margin of error.*

***Response:*** Thank you for rasing this important question. There are several difficulties that we have encountered during sample collection for this study.

A Dot commune has a total of 370 purely agricultural households, of which, 224 households (60.8%) are minority households. Za Hung commune has a total of 210 purely agricultural households with 168 households (80%) are minority households. Tra Son commune has a total of 597 purely agricultural households with 226 households (38%) are households. The total of minority households in this area are 618 households. This is a small total sample (N), so we used Yamane’s formula to calculated the sample size (n) using the following formula.

n = N/1+N.e2

Where (e) is the level of precision (5%). Based on this formula, we identified n= 243 households. However, since the investigation area is too large and difficult to reach, we were able to contact 236 households, of which, 231 households agreed participate in our survey. However, when we started our survey (at the beginning August, 2018), dozens of the households in our investigation area had moved out because of flooding, making the total number of ethnic minority households at that time only 401 households and the sample size decreased to n= 200 households.

The samples’ characteristics have now been included in the revised manuscript for better understanding.

***Revised text:*** In the present study, three minorities communes of three mountainous districts in Central Vietnam were selected for the survey. A Dot commune (A Luoi district, Thua Thien Hue province) has 132 purely minority agricultural households. Za Hung commune (Dong Giang district, Quang Nam province) has 141 purely minority agricultural households and Tra Son commune (Tra Bong district, Quang Ngai province) has 129 purely minority agricultural households. Fifty purely agricultural households in each commune were selected for the study using a random sampling method. As a result, a total of 150 households (300 people, including both husband and wife of each household) were included in this study. (Lines: 98 – 105).

***11.*** *"Line 118. As a result, a total of 400 questionnaires were collected, and only 300 satisfactory questionnaires were used for further analyses".*

*My comment -this may introduce some biases.*

***Response:*** Thank you for your suggestion. To limit the rate of homogeneity of the samples, the interview of two people in the same household was designed using before – after principle. Accordingly, the interview was done privately one immediately after the other.

Despite of our best efforts, some of our interviewers did not strictly follow this principle, thus the interview of two people in the same household was not consecutively conducted. To ensure the data accuracy, these questionnaires were excluded afterward. As a result, there are only 300/400 questionnaires were used for further data analysis.

***12.*** *My comment: Any reason for not using ordinal regression?*

***Response:*** Thank you for rasing this important question. In the present study, we used binary logistic model for 2 main reasons:

First, the people of ethnic minority area in central Vietnam are facing many problems such as poverty, low education level, unemployment, gender inequality, domestic violence, etc. In the present study, we aim to provide some policy recommendations for this area based on the outcomes. The binary regression model is a probabilistic (right/lost) predictive model which is better fit for this specific need. For these reasons, the binary regression model was applied in the present study.

Second, Weai's original index uses the cut-off index to determine the empowered rate. So we aim to use Weai's calculation to determine our binary dependent variable.

However, your valuable suggestion of using ordinal regression will certainly be considered for any of our future studies.

***13.*** *"Lines 161-163. Especially, the rate of child marriage was more than three times higher in females (23.9%) than 162 in males (7.6%). The average age of respondents was 36.1 years old, in which the average age of 163 females (37.95 yo) was higher than males (34.55 yo) (p = 0.00)"*

*My comments: Authors read the table wrongly. Females (34.6) are younger than males (38 years). Use one decimal place, instead of 2.*

**Response:** Thank you for pointing this out. The text has been edited accordingly and changes have been highlighted in red.

***Revised text:*** Notably, the proportion of child marriage (married under 18 years old) in this area was relatively high, accounted for about 15.8% of the studied populations. Especially, the rate of child marriage was more than three times higher in females (23.9%) than in males (7.6%). The average age of all respondents was 36.1 years old, in which the average age of males (38.0 yo) was higher than females (34.6 yo) (*p = 0.00*). The average number of years attended in school in both sexes combined was 8.7 years and there was a significant difference in this indicator between males and females. Accordingly, the average number of years attended in the school of males (9.6 years) was significantly higher than that of females (7.8 years) (*p = 0.00*). The average number of children was 2.9. There were no differences in the gender equality awareness index between males and females in this study (*p > 0.05*) (Table 2). (Lines: 153 – 162).

***14*** *The average number of years of children's education is 5.4 years, indicating that most are in primary schools due to their young age. It will not provide a meaningful comparison between younger and older respondents, as the latter would have older children, and hence more likely to have more years of schooling.*

***Response:*** Thank you for your comment. As we have mentioned in the answer to your comment #4, during the pilot survey (April, 2018), we found that children of ethnic minority households had years of schooling that did not match their age. This is because children in this area enter the first grade much later than those of other areas. For example, there are many children of the interviewed households who are already 14 or 15 years old but are only at 5th grade. Moreover, the percentage of students attending secondary school in this area is low. In many households, children dropped out of school halfway, or only finish first grade, or even don't go to school at all. The main reasons given are: poverty, long distance from school, language barrier. As a result, for this area, even just knowing how to read and write has already made a big difference. This finding also coincides with several other recent studies on ethnic minorities in Vietnam. Therefore, assumtion that older respondents would have older children, and hence more likely to have more years of schooling may not be correct for this specific area.

Moreover, we have tested this variable and found that it really makes sense in our regression model. This could be explained by the result of the interaction between children and parents. We will continue to test this variable in the future studies on other ethnic minorities in Vietnam and hopefully, it will be a novel and meaningful variable in policy recommendations.

***15.*** *Figures 1 and 2: My comments- Need to explain these more clearly. One can also present the different views from males and females on women's rights and empowerment. Do these figures represent the assessment by both genders, or males on males, and females on females?*

***Response:*** Figures 1 and 2 represent the self-assessment by both genders obtained from the questionnaires that used to ask both man and woman of the same household. We did not conduct a cross - review questionnaire. This can be seen in the questionnaire appended in the appendix. The legend of Fig1 has now been revised in the revised manuscript for better understanding.

***Revised text:*** Fig 1. The difference between men and women self-assessment in (A) Decision-making power and (B) Confidence level in making decision in agricultural production activities. (Lines: 190 – 191).

***16.*** *"Lines 201-201 The overall average value of participation in agricultural activities, which was found to be 0.62 in males and 0.48 in females, had further confirmed the gender differences in empowerment (p = 0.00). At the cut-off of 0.6, the loss of women's rights was significantly higher (78.1%) as compared to men (41.4%). At the cut-off of 0.8, women lose their rights completely (100%) while that proportion in men was 89% (Fig 2A).*

*My comment: It will be more appropriate to state that "with a cut-off of 0.6, only 21.9% of the women achieved this threshold, compared to 58.4% of the males. None of the women had achieved a threshold of 0.8 compared to 11% of the males. The same suggestion applies to other parts of the article.*

***Response:*** Thank you for the valuable comment. The content has now been revised throughout the revised manuscript as per your suggestion and changes have been highlighted in red.

***Revised text:***

- With a cut-off of 0.6, only 21.9% of the women achieved this threshold, compared to 58.4% of the men. None of the women had achieved a threshold of 0.8 compared to 11% of the men (Fig 2A) (Lines: 194 – 195).

- With a cut-off of 0.6, only 5.5% of the women achieved this threshold, compared to 60.6% of the men. 5.5% of the women had achieved a threshold of 0.8 compared to 66.5% of the men (*p = 0.00*) (Fig 2C). (Lines: 245 – 247).

- There was no statistically significant difference between men and women in the social participation. With a cut-off of 0.6, 21.9% of the women achieved this threshold, compared to 27.9% of the men (*p > 0.05*). Similarly, at the cut-off of 0.8, there was no also statistically significant difference. (Fig 2D). (Lines: 259 – 262).

- With a cut-off of 0.6, only 29.4% of the women achieved this threshold, compared to 41.2% of the men. 1.4 % of the women had achieved a threshold of 0.8 compared to 23.7% of the men (*p = 0.00*) (Fig 2E). (Lines: 269 – 271).

- The general assessment of the above 5 indicators revealed a big difference in the empowerment between men and women (*p = 0.00*). With a cut-off of 0.6, only 30.3% of the women achieved this threshold, compared to 84.8% of the men. Only 19.4% of the women had achieved a threshold of 0.8 compared to 53.8% of the men (*p = 0.00*) (Fig 2F). (Lines: 273 – 276).

***17.*** *Need to make reference to tables 3 and 4 in the text. The description is rather confusing, and it is very hard to relate the numbers mentioned in the text and the tables. Need to write more clearly. Show statistical significance.*

***Response:*** Thank you for the suggestion. This part has now been rewritten as per your suggestion and changes have been highlighted in red.

***Revised text:*** Resource ownership was assessed by three indicators, namely (i) property ownership; (ii) to access loans, and (iii) decision-making power in borrowing capital.

For content (i), our results revealed a substantial difference in the property ownership between males and females (*p = 0.00*), especially in high value assets. For example, up to 52.4% of men thought that agricultural land was owned by themselves, while, none of the women said that they owned this type of asset (0%). For other assets such as cattles, farm equipment, non-agricultural land, houses, and durable goods, the proportion of men who chose the answer of owning it alone (52.4, 64.1, 35.2, 64.1, and 64.2%, respectively) was substantially higher than that of women (6.1, 0.0, 0.0, 6.1, and 6.1%, respectively) (Table 3).

For the ability to access loans, there was a statistically significant difference between males and females (*p = 0.03*). Accordingly, in the forms of formal and informal credits, the percentage of female respondents answering that they definitely could borrow money was significantly lower than men (formal credit: 34.7% vs. 59.3%; informal credit: 40.8% vs. 55.2%). Only in the form of non-government organizations (NGOs) credit, this rate was higher in women than men (17.7% vs. 11%) but this difference was not significant (*p = 0.07*) (Table 4).

Regarding decision-making power in borrowing activities (iii), similar to the above two contents, there is also a big difference between men and women. In formal credit institutions, 24.1% of men said that they make their own decisions when borrowing while women are only 0%. This ratio in the form of informal credit is 24.8% for men, compared to 6.1% for women (Table 4). There was no gender difference in decision making for borrowing from NGOs (*p > 0.05*) (Table 4). (Lines: 204 – 224).

***18.*** *For instance, "The overall average value of the ownership of the resources had also shown the gender difference, with values of 6.1/10 for men and 4.4/10 for women (p = 0.00) - I cannot find these figures in the tables.*

***Response:*** This was the average score before we applied the cut-off index to determine the empowerment threshold. To avoid confusing the readers, we have now remove these values from the revised manuscript and text has been revised accordingly.

***Revised text:*** The overall value of the ownership of the resources had also shown the gender difference. Only 40% of the women achieved a cut-off 0.6 compared to 61.3 % of the men. with a cut-off of 0.8, with approximately 85% for both sexes do not achieve this threshold (*p > 0.05*) (Fig 2B). (Lines: 225 – 227).

***19.*** *Table 5 under this sub-section shows decision-making on income generation, not on the use of income -wrong label?*

***Response:*** Thank you for pointing this out. The label of Table 5 has been edited based on your comment.

***Revised text:*** Table 5. Gender differences on control over the use of income. (Line: 248)

***20.*** *Ordinal regression may be a good alternative. Explain why this was not used. Authors may present a cross-tab to show the average score or percent with a score of 0.6 and over by the independent variables, and conduct statistical test. The age factor may be significant at the bivariate level, but its effect is explained away by number of children. The effect of age at first marriage may be attenuated after adjusting for other variables in the model. Show crude odds and adjusted odds ratiosCategorical independent variables (recoded from the ratio scale variables) - may show the effects of these variables more clearly (the significant differences across the various categories).*

***Response:*** Thank you for bringing this up again. As we have mentioned on the answer to your comment #12, we used binary logistic model for 2 main reasons:

First, the people of ethnic minority area in central Vietnam are facing many problems such as poverty, low education level, unemployment, gender inequality, domestic violence, etc. In the present study, we aim to provide some policy recommendations for this area based on the outcomes. The binary regression model is a probabilistic (right/lost) predictive model which is better fit for this specific need. For these reasons, the binary regression model was applied in the present study.

Second, Weai's original index uses the cut-off index to determine the empowered rate. So, we aim to use Weai's calculation to determine our binary dependent variable.

Moreover, during the design of the survey, we used WEAI's measurement framework, and from the outset, intentions were calculated for a probabilistic regression model. So, if we changed the model at this point, we would have to change most of the results we presented in the paper. We know that the critique's suggestion for an OLS regression model is valuable, and we will surely apply it in our future studies.

***21.*** *Figures 2A - 2F - Label the figures.*

***Response:*** Thank you for your comment. The detailed labels for Figures 2A-2F have now been included in the revised manuscript.

***Revised text:*** Fig 2. The rate of reach the threshold of empowerment of male and female at cut-off values of 0.6 and 0.8 in (A) Participation in agricultural production activities; (B) Own resources; (C) Control over the use of income; (D) Social participation; (E) Time use; (F) General empowerment in agriculture. (Lines: 197 - 200).

***22.*** *"Line 346 loss of credit decisions...."*

*My comment - This again assume that the women had the autonomy to make credit decision or access credit, but lost it"*

***Response:*** Thank you for your comment. The content has now been revised in the revised manuscript.

***Revised text:*** Empowerment studies in developing countries has commonly been reported, women have less power than men in credit-related decisions [34,35]. In concordance with that, our study also showed women have much less power than men in credit-related decisions. This observation can be explained by two main reasons: (i) in developing countries, the majority of the householders were men, who were legally recognized and had the right to own valuable assets. Therefore, they also have the top authority to mortgage these assets for loans; (ii) cultural, legal, and economic barriers, the level of financial awareness, or differences in gender behaviour also hindered women's decisions on this issue (Lines: 329 - 336).

***23.****"Line 355-356 ....number of previous studies in Vietnam and other developing countries, however, claimed that women were whom to hold money and to decide how to use them [10,11"]*

*My comments: Most studies found that women and men make joint decision, not sole decision on household spending and on buying propoerties.*

***Response:*** This sentence has now been revised as per your suggestion.

***Revised text:*** Our results also show that men are still the main decision makers in household expenditure which is different from most studies showing that women and men make joint decisions on household expenditures [10,11]. (Lines: 337 - 339).

***24.*** *"Line 372-375 As mentioned above, due to men’s chauvinism, few men volunteered to do housework with their wives. They often shun the housework and considered it a women's job. This is also the main reason why women do not have much rest and leisure time to recreate labor power".*

*My comment: More like gender role stereotyping, not so much male chauvinism.*

***Response*:** This part has now been rewritten as per your suggestion. Changes have been highlighted in red.

***Revised text:*** Women in the research areas, apart from participating in farming, also must undertake all the housework and taking care of the family (the unpaid works). As mentioned above, due to gender role stereotyping, few men volunteered to do housework with their wives. They often shun the housework and considered it as a women's duty. This is also the main reason why women do not have much rest and leisure time to recreate labor power. (Lines: 353 - 358).

***25.****"Lines 382-383. ...most of the empowerment studies [18,20–22,24,34], the age at first marriage, the highest education of children detected in this study were novel factors..."*

*My comments: Women's age at marriage is closely related to educational level.*

***Response:*** Thank you for your comment. The sentence has now been revised accordingly.

***Revised text:*** While the education level, the distance to the nearest urban area, and the number of children is related to women's empowerment were the common findings in most of the empowerment studies [18,20–22,24,34], the age at first marriage, the highest education of children detected in this study were novel factors. In particular, the age of first marriage is closely related to empowerment. (Lines: 363 - 367).

***26.*** *"Lines 396-397. Firstly, in the present study, we found that among the five assessed factors, the rate of women's disempowerment was significantly higher than that of men."*

*My suggestion: Women in rural agricultural setting were played a lesser role than the men, across socio-demographic variables.*

***Response:*** This has now been rewrite as per your suggestion. Changes have been highlighted in red as the following.

***Revised text:*** Firstly, in the present study, we found that in all five assessed factors women in rural agricultural setting were played a lesser role than the men, across socio-demographic variables. Resource ownership, spend time, and household financial control were are typical elements. (Lines: 379 - 381).

**Thank you very much for all your valuable comments and suggestions!**

19 October, 2022

To

Reviewer #2

Dear Reviewer,

We would like to thank the reviewer for the insightful review and valuable comments on our manuscript. In the revised version, we hope that we have satisfactorily addressed the comments raised by the reviewer.

Here below, we provide a point-to-point answer to all remarks. All the changes have been highlighted in the manuscript in red and a clean version with line numbers was also provided.

We would like to thank the reviewer again for taking the time to review our manuscript. We hope the manuscript after careful revisions meet your high standards. We are happy to respond to any further questions and comments you may have.

Your sincerely,

Thanh Tung Le

On behalf of all authors

**Reviewer #2**

***I. Major issues***

***1.*** *My main concern has to do with the methodology for constructing the empowerment measure and the interpretation of empowerment.* *Confusion regarding the WEAI – The paper presents the WEAI as the tool used for measuring empowerment, but it actually does not use the WEAI. The domains and indicators may be called the same but the questions and response options are different. All the questions used Likert scales, and then the authors apply the notion of a cutoff to each individual indicator. In the WEAI, the cutoff is used to identify empowered individuals (ie, those who achieve 4 out of 5 domains or 80% of the weighted indicators are defined as empowered), whereas individual indicators each have separately defined thresholds that were informed by both theory and validation analyses. This means that the resulting indicators and composite index presented in the paper does not inherit any of the (validated) properties of the WEAI itself. Rather this is a whole new index, despite using the same domain/indicator headings and weights. At best this is “inspired” by the WEAI or is using the WEAI only as a “framework”, rather than using the WEAI methodology and tool itself. I suggest that the paper be very explicit in exactly which aspects of the WEAI methodology are used, and which parts have been modified. Otherwise readers will incorrectly assume this is using the same standardized WEAI methodology. I also suggest calling this empowerment index a different name all together to avoid confusion, and perhaps simply cite WEAI for the parts that are actually used in the paper.*

***Response:*** Thank you for your valuable comment. We agreed that instead of using WEAI as a measuring tool, we used it as a reference. We have revised and clearly stated this point in the revised manuscript. Additionally, we have also mentioned that we inherit the variables and the way we calculate the weights to build the questionnaire for our research. Changes have been highlighted in red.

***Revised text:***

- We used The United States Feed for Future Assessment (WEAI) as a framework for reference. By inheriting the empowerment indicators and calculations of WEAI, we designed a structured questionnaire to interview 300 people including both men and women in the same household. (Lines: 32 - 35).

- In the present study, we used The United States Feed for Future Assessment (WEAI) as a framework for reference. By inheriting five domains of empowerment (5DE) of WEAI: (i) decisions on agricultural production, (ii) own and make decisions about resources, (iii) control and use of income, (iv) social participation, and (v) the use of time; we designed a structured questionnaire to interview 300 people including both men and women in the same household [3]. We also inheriting WEAI's calculations to calculate final empowerment index (EI) is the weighted average of the 5DE (Table 1). The degree of empowerment ranged from 0 to 1. Individuals reach the threshold of empowerment if he/she has the final EI larger than the cut-off value of 0.8 [3]. (Lines: 84 - 91).

***2.*** *Empirical strategy****.*** *Given that all the indicators are collected as Likert scales, converting these into binary indicators using an arbitrary cutoff is a waste of information. Instead, I suggest simply adding up the variables to come up with an aggregate scale and then use OLS to run the regressions. The use of thresholds and cutoffs in the WEAI is important for decomposition, which is no longer relevant in this case since the data (Likert scales) will not allow for WEAI-style decomposition. There are a growing number of studies that use WEAI indicators that are moving beyond simply the binary indicators (for example, Quisumbing et al 2021:*

[*https://doi.org/10.1016/j.foodpol.2020.102001*](https://doi.org/10.1016/j.foodpol.2020.102001)*). In my view this is a more appropriate indicator to use for regression analysis because it does not depend on the choice of cutoff, which the paper also points to as problematic.*

***Response:*** Thank you for your suggestion. We agree that OLS model may help to avoid the choice of cutoffs which is required while using binary regression. However, during survey design and data collection, we consulted WEAI's measurement framework and variables were collected for a probabilistic regression model. So, if we changed the model, we would have to change most of the results presenting in the paper.

***Response:*** Thank you for rasing this important question. In the present study, we used binary logistic model for 2 main reasons:

First, the people of ethnic minority area in central Vietnam are facing many problems such as poverty, low education level, unemployment, gender inequality, domestic violence, etc. In the present study, we aim to provide some policy recommendations for this area based on the outcomes. The binary regression model is a probabilistic (right/lost) predictive model which is better fit for this specific need. For these reasons, the binary regression model was applied in the present study.

Second, Weai's original index uses the cut-off index to determine the empowered rate. So, we aim to use Weai's calculation to determine our binary dependent variable.

However, your valuable suggestion of using ordinal regression will certainly be considered for any of our future studies.

***3.*** *Regarding the empirical specification, I wonder whether children’s education is simply picking up the age of the kids*

***Response:*** Thank you for your question. The children’s education is counted as the number of years of schooling. For example, a student who has finished 5th grade will be counted as 5 years of schooling.

During the pilot survey (April, 2018), we found that children of ethnic minority households had years of schooling that did not match their age. This is because children in this area enter the first grade much later than those of other areas. For example, there are many children of the interviewed households who are already 14 or 15 years old but are only at 5^th^ grade. Moreover, the percentage of students attending secondary school in this area is low. In many households, children dropped out of school halfway, or only finish first grade, or even don't go to school at all. The main reasons given area: poverty, long distance from school, language barrier. As a result, for this area, even just knowing how to read and write has already made a big difference. This finding also coincides with several other recent studies on ethnic minorities in Vietnam.

We have tested this variable and found that it really makes sense in our regression model. This could be explained by the result of the interaction between children and parents. We will continue to test this variable in the future studies on other ethnic minorities in Vietnam and hopefully, it will be a novel and meaningful variable in policy recommendations.

***4.*** *I would also suggest using number of young children (aged <5yo) instead of number of children overall, since the care needs of young children are much greater and would arguably be more disempowering*

***Response:*** Thank you for your suggestion. However, this specific piece of information was not included in the current study study design and questionnaire; thus, this variable could not be included in the current study.

***5.*** *Interpretation of empowerment – I was quite confused by the paper’s references to “loss of rights” throughout the paper. WEAI is primarily measuring agency, and it is indeed possible to have agency even when rights are not guaranteed. While there are indicators that can be interpreted as "rights over assets" in the WEAI, this does not extend to the whole of the empowerment index. In the WEAI’s control over income indicator, those questions are phrased as inputs over income decisions, whereas for this paper, it appears that the response options include mention of rights, but it is not clear whether that is appropriate (I would suggest sharing the questionnaire also in an Appendix). For example, the concept of rights over workload to me does not make sense, especially since the response options there appear to be a range of work hours. Rights are quite a different concept from empowerment even though they are related. I would suggest sticking to the empowerment definition that the authors refer to in lines 43-44 which is really referring to agency or the power to make important decisions.*

***Response:*** Thank you for the suggestion. The term “lose empowerment” has now been changed as per your suggestion throughout the revised manuscript. Changes have been highlighted in red as the following.

***Revises text:***

- The results showed that up to 70% of women did not reach the threshold of empowerment (Lines: 37 – 38).

- These studies showed that women often generally have fewer rights than men to property ownership, including land, capital, and durable goods (Lines: 63 – 65).

- With a cut-off of 0.6, only 21.9% of the women achieved this threshold, compared to 58.4% of the men. None of the women had achieved a threshold of 0.8 compared to 11% of the men (Fig 2A) (Lines: 194 - 195).

- With a cut-off of 0.6, only 5.5 % of the women achieved this threshold, compared to 60.6% of the men. 5.5% of the women had achieved a threshold of 0.8 compared to 66.5 % of the men (p = 0.00) (Fig 2C). (Lines: 245 - 247).

- There was no statistically significant difference between men and women in the social participation. With a cut-off of 0.6, 21.9 % of the women achieved this threshold, compared to 27.9 % of the men (p > 0.05). Similarly, at the cut - off of 0.8, there was no also statistically difference significant. (Fig 2D) (Lines: 259 - 262).

- With a cut-off of 0.6, only 29.4 % of the women achieved this threshold, compared to 41.2 % of the men. 1.4 % of the women had achieved a threshold of 0.8 compared to 23.7 % of the men (p = 0.00) (Fig 2E) (Lines: 269 – 271).

- The general assessment of the above 5 indicators revealed a big difference in the empowerment between men and women (p = 0.00). With a cut-off of 0.6, only 30.3 % of the women achieved this threshold, compared to 84.8 % of the men. Only 19.4 % of the women had achieved a threshold of 0.8 compared to 53.8 % of the men (p = 0.00) (Fig 2F) (Lines: 273 – 276).

- Because the percentage of both men and women not reaching the empowerment threshold of 0.8 is very high (Lines: 286 – 287).

- In addition, our results also indicated that most of the women in the rural area have less rights than men of property ownership, especially for valuable properties such as farm equipment and non-agricultural land (Lines: 322 – 324).

- Firstly, in the present study, we found that in all five assessed factors Women in rural agricultural setting were played a lesser role than the men. Resource ownership, spend time, and household financial control were are typical elements. (Lines: 379 – 381).

- Secondly, 70% of women in this study didn't reach the empowered threshold in agriculture signified a large gender gap in these specific geographic areas, and suggested an urgent need for specific and timely policy interventions to increase women's empowerment in these regions (Lines: 382 – 384).

***II. Minor issues:***

***6.*** *The paper is based on observational data and therefore the regression estimates should not be interpreted as causal relationships. I suggest avoiding all mentions of “impact” and other causal language, such as in lines 311, 390, and 425.*

***Response:*** Thank you for the suggestion. All of this content has now been changed as per your suggestion throughout the revised manuscript. Changes have been highlighted in red as the following.

***Revises text:***

- Ethnic minority women’s empowerment in agriculture in the central region of Viet Nam and its related factors. (Lines: 4 – 5).

- The results showed that up to 70% of women did not reach the threshold of empowerment. The regression analyses using the binary logistic model revealed the age at first marriage, the level of children's education, education level, distance to the nearest urban area, and the number of children were related on the probability of empowerment. Age, income, and the level of gender awareness did not show any related. (Lines: 37 – 42).

- Evaluate the factors related to empowerment by the logistic regression model (Lines: 277).

- To evaluate the empowerment’s related factors, different regression models have been tested. However, the binary logistic regression model has appeared to be the most suitable model for the following reasons: (Lines: 278 – 280).

- Table 6. Regression results factors that related the probability of empowerment. (Lines: 307).

- In general, our results reflected the reality of gender differences in empowerment in agriculture-related activities in the survey areas. Particularly, the assessment of the related factors of empowerment would be an important basis for proposing policies that seek to boost the level of women's empowerment in agriculture. Similar to the previous studies in Vietnam [26], our results also showed the absolute control of men on agricultural production-related decisions (Lines: 314 - 318).

- Empowerment studies in developing countries has commonly been reported, Women have less power than men in credit-related decisions [34,35]. In concordance with that, our study also showed Women have much less power than men in credit-related decisions. (Lines: 329 – 331).

- In particular, the age of first marriage is closely related to empowerment (Lines: 366 – 367).

- The remained factors such as age, income, and awareness of gender equality had no related on the level of empowerment. (Lines: 376 - 377).

***7:*** *Please provide clarifications on the following:* *27-28: refers to “composite index” in the opening line, but not clear what is being referred to here*

***Response:*** The composite index that we mean here is the average empowerment index of the component factors. This content has now been included in the revised manuscript for better understanding.

***Revises text:*** Gender inequality and empowerment are two closely related issues. While the Gender Inequality Index has been assessed by different studies, that of empowerment remained to be very limited. In the present work, we attempt to measure the empowerment index for ethnic minority women in agriculture in Central Vietnam. (Lines: 29 - 32).

***8.*** *35-36: not sure what this means: “70% lose their empowerment in their final empowerment index”*

***Response:*** Thank you for the comment. As we answered in comment 5, the term “lose empowerment” has now been changed as per your suggestion throughout the revised manuscript. This sentence has now been revised accordingly.

***Revises text:*** The results showed that up to 70% of women did not reach the threshold of empowerment. (Lines: 37 - 38).

***9.*** *47-50: very long sentence, hard to read; suggest putting the long lists in parentheses, or splitting this up for claritys*

***Response:*** Thank you for the comment, This sentence has now been revised accordingly.

***Revises text:*** Empowerment is the ability of a group or individual to make decisions involved in resource ownerships and typical activities [1,2]. Accordingly, empowerment in agriculture is defined as the ability to own and make decisions of a group or individual on issues related to agricultural resources [3], including: ownership for valuable resources; decisions on time usage and income usage; and the level participate in social organizations [4]. (Lines: 45 - 49).

***10.*** *“constant variables” – constant in what sense?*

***Response:*** The constant variables we were talking about is the most commonly cited variables. Changes have been highlighted in red as the following.

***Revised text:*** A number of the previous studies using a Logit regression model to assess the probability of empowerment-related factors revealed that educational attainment and household income were the most commonly cited variables. (Lines: 68 – 70).

***11.*** *91-92: is EI referring to an individual-level empowerment measure or is it like the WEAI which is reported at the program/sample level so it does not vary across individuals?*

***Response:*** Thank you for your question. We want to conduct a program- or large-sample empowerment measurement study to compare differences across geographies. However, gender research in a special geographical area such as the ethnic minority area in central Vietnam is very difficult. Therefore, in this study, we only aimed to assess empowerment at the individual level. Even so, we are still particularly looking forward to being able to do a larger level study. If there are enough resources, we will implement it as soon as possible.

***12.*** *101-102: it would be useful to give more context to why this particular region was selected.*

***Response:*** Thank you for rasing this important question. There are several difficulties that we have encountered during sample collection for this study.

A Dot commune has a total of 370 purely agricultural households, of which, 224 households (60.8%) are minority households. Za Hung commune has a total of 210 purely agricultural households with 168 households (80%) are minority households. Tra Son commune has a total of 597 purely agricultural households with 226 households (38%) are households. The total of minority households in this area are 618 households. This is a small total sample (N), so we used Yamane’s formula to calculate the sample size (n) using the following formula.

n = N/1+N.e2

Where (e) is the level of precision (5%). Based on this formula, we identified n= 243 households. However, since the investigation area is too large and difficult to reach, we were able to contact 236 households, of which, 231 households agreed participate in our survey. However, when we started our survey (at the beginning August, 2018), dozens of the households in our investigation area had moved out because of flooding, making the total number of ethnic minority households at that time only 401 households and the sample size decreased to n= 200 households.

The samples’ characteristics have now been included in the revised manuscript for better understanding.

***Revised text:*** In the present study, three minorities communes of three mountainous districts in Central Vietnam were selected for the survey. A Dot commune (A Luoi district, Thua Thien Hue province) has 132 purely minority agricultural households. Za Hung commune (Dong Giang district, Quang Nam province) has 141 purely minority agricultural households and Tra Son commune (Tra Bong district, Quang Ngai province) has 129 purely minority agricultural households. Fifty purely agricultural households in each commune were selected for the study using a random sampling method. As a result, a total of 150 households (300 people, including both husband and wife of each household) were included in this study. (Lines: 98 – 105).

***13.*** *Is the WEAI data collected part of a larger study?*

***Response:*** Thank you for your question. This data is part of Duy Tan University's project "research some issues on rural ethnic minorities in Central Vietnam". This project focuses on research on household economic restructuring, gender inequality, and domestic violence.

***14.*** *Table 2: In general, household level characteristics and individual level characteristics should be presented separately. I am curious to know why distance is the only HH level characteristic of interest?*

***Response:*** Thank you for rasing this important question. Distance from respondents' houses to the nearest urban area was not the only household characteristic variable. We also have the variable household income/month. We have added it as follows.

***Revises text:***

| **Research sample** | **Mean** ± **SD** | | |
| --- | --- | --- | --- |
|  | **Total** | **Male** | **Female** |
| **Personal characteristics** | | | |
| **Gender** | 100% | 48.3% | 51.7% |
| **Age** | 36.0 ± 9.2 | 38 ± 8.8*** | 34.5 ± 9.4 |
| **Education** | 8.7 ± 2.8 | 9.4 ± 2.5*** | 7.8 ± 2.8 |
| **The gender equality awareness index** | 3.7 ± 1 | 3.6 ± 1 | 3.7 ± 1.1 |
| **Age at first marriage** | 20 ± 2.9 | 20.7 ± 2.7*** | 19.3 ± 3 |
| **Household characteristics** | | | |
| **Distance from respondents' houses to the nearest urban area** | 19.1 ± 9.7 | NA | NA |
| **Income** | 3.8 ± 1.6 | NA | NA |
| **Number of children** | 2.9 ± 1.2 | NA | NA |
| **Education of children** | 5.3 ± 3.7 | NA | NA |

- The average income per household/month is very low, only 3.8 million VND (about 200 USD/month). (Lines: 150 – 151)

***15.*** *221: is this really zero? if so better to say in the text that none of the women said xyz*

***Response:*** Thank you for the comment, This sentence has now been revised accordingly.

***Revises text:*** For content (i), our results revealed a substantial difference in the property ownership between males and females (p = 0.00). Especially in high value assets. For example, up to 52.4% of men thought that agricultural land was owned by themselves, while, none of the women said that she owned this type of asset (0%). (Lines: 206 – 209)

***16.*** *325: Table 6: is this for women only? Table should note the number of observations*

***Response:*** Thank you for your question. This table is a regression table for women. We have added the number of observations to the table's footnote. Changes have been highlighted in red as the following:

***Revises text:*** B: Estimated coefficient; S.E: standard error of the mean; Wald: wald test meaning; Df: the degrees of freedom coefficients; p value: the level of statistical significance; Exp(B): estimated coefficient weights; number of observations: 155. (Lines: 308 – 310).

***17.*** *345: maybe “male chauvinism” instead of “men chauvinism”?*

***Response:*** Thank you for the comment. To make it easier to understand, we have replaced the phrase "male chauvinism" with the phrase "gender role stereotyping". Changes have been highlighted in red as the following:

***Revised text:*** Women in the research areas, apart from participating in farming, also must undertake all the housework and taking care of the family (the unpaid works). As mentioned above, due to gender role stereotyping, few men volunteered to do housework with their wives. They often shun the housework and considered it as a women's duty. This is also the main reason why women do not have much rest and leisure time to recreate labor power. (Lines: 353 - 358).

***18.*** *340-341: “lose their rights of property ownership”: lose because of what?*

***Response:*** Thank you for the comment, the term “lose empowerment” has now been changed as per your suggestion throughout the revised manuscript. This sentence has now been revised accordingly:

***Revises text:*** In addition, our results also indicated that most of the women in the rural area have less rights than men of property ownership, especially for valuable properties such as farm equipment and non-agricultural land (Lines: 322 – 324).

***19:*** *390: “interaction process”: what interaction process?*

***Response:*** Thank you for your question. “interaction process” are the daily interactions in the family. This content has now been revised accordingly:

***Revises text:*** It was believed that the daily interactions in the family has changed the perception of family members, especially the right of a mother to participate and decide different family issues. (Lines: 373 – 375).

***20.*** *399-400: “seventy percent of women in this study lost power in the final EI signified a large gender gap”: I don’t understand this point?*

***Response:*** Thank you for your question. The term “lose power” has now been changed as per your suggestion throughout the revised manuscript. This sentence has now been revised accordingly.

***Revises text:*** 70% of women in this study didn't reach the empowered threshold in agriculture signified a large gender gap in these specific geographic areas, and suggested an urgent need for specific and timely policy interventions to increase women's empowerment in these regions (Lines: 382 – 384).

***21.*** *429-432: I agree that using mixed methods is better than using only quant or qual tools by themselves. There are now qualitative protocols that are recommended for use along with the pro-WEAI for future work which I think is worth acknowledging:* [*https://weai.ifpri.info/weai-resource-center/guides-and-instruments/*](https://weai.ifpri.info/weai-resource-center/guides-and-instruments/)*.*

***Response:*** We especially thank the reviewer for this suggestion. We are trying to combine qualitative and quantitative research methods in subsequent studies on empowerment in ethnic minority areas, central Vietnam.

***22.*** *There are a number of typos in the document. If the authors have the resources, I would also strongly encourage hiring a professional editor to go over the language of the paper to improve clarity and readability of the paper. Please note that PLOS ONE does not provide copyediting.*

*21. 75: typo, should be “extent”*

*22. 107: typo, should be “Likert”*

*23. typo, should be “Fifthly”*

***Response:*** Thank you for pointing these out. All spelling errors have now been corrected in the revised manuscript. The manuscript has also been proofreaded by two researchers with extensive experience in completing manuscripts for publication. I truly hope that the manuscript is now meet the standard of PLOS ONE.

**Thank you very much for all your valuable comments and suggestions!**
