# Supplementary material for: Ethnic minority women’s empowerment in agriculture in the central region of Viet Nam
Source: PLoS One. 2023 Aug 7;18(8):e0287115. doi: 10.1371/journal.pone.0287115 (PMC10406194; doi:10.1371/journal.pone.0287115)
Supplement: Attachment — Submitted filename: TNP2.doc [file pone.0287115.s004.doc]

Ethnic minority women’s empowerment in agriculture and its related factors in the central region of Viet Nam

Tung Thanh Le^1^*, Son Van Phan^2^, Duong Thanh Nguyen^3^, Huong Thi Hoang^3^, Lam Trong Vu^4,^*, Anh Tuan Nguyen^5^ , Tuyet Thi Nguyen^6^

^1^ Faculty of Social Sciences and Humanities, Duy Tan University, Da Nang 550000, Viet Nam

^2^ Faculty of Information Technology, Duy Tan University, Da Nang 550000, Viet Nam

^3^Institute of Hospitality and Tourism, Duy Tan University, Da Nang 550000, Viet Nam

^4^National Political Publishing House, Ha Noi 100000, Viet Nam

^5^Faculty of Accounting, Duy Tan University, Da Nang 550000, Viet Nam

^6^ Institute of Hospitality and Tourism, Duy Tan University, Da Nang 550000, Viet Nam

*Corresponding authors

E-mails: lethanhtung4@dtu.edu.vn (TTL); lamvutrong99@gmail.com (LTV)

**Abstract**

Gender inequality and women's empowerment are two closely related issues. While the Gender Inequality Index has been assessed by different studies, that of women's empowerment remains ~~to be very~~ limited. In the present work, we attempt to measure the women's empowerment index for ethnic minority ~~women~~ in agriculture in Central Vietnam. We used The United States Feed for Future Assessment (WEAI) as a framework for reference. By using ~~inheriting~~ the empowerment indicators and calculations of WEAI, we designed a structured questionnaire to interview 300 people including both men and women in the same household. The difference in the empowerment level between men and women was assessed ~~identified~~ through five-component empowerment indicators: agricultural participation, resource ownership, financial control, social organizations participation, and time usage.The results showed that up to 70% of women did not attain ~~reach~~ the threshold of empowerment. ~~The regression analyses using~~ the binary logistic model revealed the age at first marriage, the level of children's education, education level, distance to the nearest urban area, and the number of children were related ~~on the probability of~~ women's empowerment, but age, income, and the level of gender awareness were not ~~did not show any related.~~

**Introduction**

Empowerment is the ability of a group or individual to make decisions involved in resource ownerships and typical activities [1,2]. Accordingly, empowerment in agriculture is defined as the ability ~~of a group or individual~~ to own ~~on issues related to~~ agricultural resources ~~[3],~~ and make decisions ~~including: ownership for valuable resources; decisions~~ on time usage and income usage; and the level participate in social organizations [3, 4].

A study conducted recently in Ethiopia in Africa showed that eighty-five percent of the population is concentrated in the agricultural sector, with ~~of which~~ women ~~are~~ accounting for nearly ~~a~~ half [5].However, studies indicate that women in agriculture still face gender inequality~~, including gender inequality~~ in: property ownership, right of financial access, social participation, and time usage [2,6,7]. These gender gaps can reduce household income and access~~ibility~~ to basic social services such as education and healthcare~~, etc~~. [8]. Therefore, women's empowerment in agriculture is considered one of the crucial factors ensuring global food security and promoting human development goals [2,6,9].

Although the role of women's empowerment in development has been addressed in several theoretical studies, ~~the number of~~ empirical research~~es~~ on this topic is still limited. In most of the available empirical studies in rural areas, women's empowerment was often studied in a single aspect, such as economic empowerment, political empowerment, or property ownership ~~empowerment~~; and very few studies have measured the composite empowerment index. One of the main reasons for this could be the difficulty in the measurement framework set-up [3,10–13]. These studies showed that women ~~often~~ generally have fewer rights than men to property ownership, including land, capital, and durable goods[14]. It was also more difficult for them to access credit and social participation than men [3,15]. ~~At the national level,~~ women in ~~developing countries~~ in Southeast Asia generally have ~~had~~ more rights in agricultural production decisions and control of household income than ~~as compared to~~ those in other developing regions [16]. A number of ~~the~~ previous studies using ~~a~~ Logit regression model to assess ~~the probability of~~ empowerment-related factors revealed that educational attainment and household income were the most commonly cited variables [17–24]. Besides, age, level of gender equality, and religious awareness were noted to be significantly correlated with women's empowerment [17,19], and some other exogenous factors such the quality of family relationships, housing area, non-farm employment, and the distance to the nearest urban areas to ~~with~~ a lesser extent [18].

~~In Vietnam, unlike~~ the gender inequality in rural areas and ethnic minority ~~that~~ has been well studied. However, ~~the~~ women's empowerment-related issues in Vietnam ~~in these areas~~ have only been studied recently, mostly on ~~involved in~~ economic and political empowerment. Most of the published work on women's empowerment showed that women often have less opportunity to make decisions on their life-related issues. In many cases, women are also victims of discrimination and gender violence. The main reasons for this could be due to cultural barriers, low educational attainment, lack of opportunities to own productive resources such as land, capital and property~~, etc~~. [25]. Noticeably, there are no studies in Vietnam that have measured the overall index of women's empowerment in agriculture till date.

In the present study, we used The United States Feed for Future Assessment (WEAI) as a framework for reference. ~~By~~ Using the ~~inheriting~~ five domains of empowerment (5DE) of WEAI: (i) decisions on agricultural production, (ii) own and make decisions about resources, (iii) control and use of income, (iv) social participation, and (v) the use of time; we designed a structured questionnaire to interview 300 people including both men and women in the same household [3]. We follow ~~also inheriting~~ WEAI's method ~~calculations to~~ calculate final empowerment index (EI), ~~is~~ the weighted average of the 5DE (Table 1). The degree of empowerment ranged from 0 to 1. Individuals reach the threshold of empowerment if he/she has the final EI larger than the cut-off value of 0.8 [3].

**Table 1. The WEAI’s five domains of empowerment (5DE), corresponding ten indicators, and their respective weights [3].**

| **Five domains** | **Ten indicators** | **Weight** |
| --- | --- | --- |
| **Production** | 1. Input in productive decisions | 1/10 |
|  | 2. Autonomy in production | 1/10 |
| **Resources** | 3. Ownership of assets | 1/15 |
|  | 4. Purchase, sale, or transfer of assets | 1/15 |
|  | 5. Access to and decisions on credit | 1/15 |
| **Income** | 6. Control over use of income | 1/5 |
| **Leadership** | 7. Group membership | 1/10 |
|  | 8. Speaking in public | 1/10 |
| **Time use** | 9. Workload | 1/10 |
|  | 10. Leisure | 1/10 |

**Materials and methods**

**Research data**

In the present study, three minorities communes of three mountainous districts in Central Vietnam were selected for the survey. A Dot commune (A Luoi district, Thua Thien Hue province) has 132 purely minority agricultural households. Za Hung commune (Dong Giang district, Quang Nam province) has 141 purely minority agricultural households and Tra Son commune (Tra Bong district, Quang Ngai province) has 129 purely minority agricultural households. Fifty purely agricultural households in each commune were selected for the study using a random sampling method. ~~As a result,~~ a total of 150 households (300 people, including both husband and wife of each household) were included in this study.

The interviews were carried out from March to November 2018.The data collection process was divided into three stages. The first phase was performed from March to May 2018. During this period, we conducted pilot surveys on-site training of enumerators and then modified ~~adjusted~~ the questionnaire. Similar to many other ~~gender~~ surveys, despite being well-trained, the interviewers still encountered difficulties in conducting and controlling the interview content. Consequently, more than half of the interviewers were eliminated after executing the first trial interview. The second phase was run from June to September 2018. For the effectiveness of data collection, we decided to include enumerators with experience in the sociological survey to collect the data. The third phase was the last two months, from October till the end of November of that year. During this period, we finalized the administrative work and checked the final data.

This study is part of the socio-economic research project on the rural areas of the South-central coast of Vietnam. All the questionnaires were stamped and signed by the implementing agency. All the respondents agreed to participate in this project and answered the questionnaire.

**Methods**

**Data encoding**

For consistency and convenience of data analysis, we adopted ~~inherited~~ five elements of empowerment with ten indicators of WEAI. The answer’s options for questions in each field were coded according to the Likert scale, and coded ~~attributed~~ with a number from 1 to 5, as follows: 1 = absolutely no right in decision-making; 2 = decide with someone who is not a family member; 3 = decide with a non-spouse family member; 4 = decide with the spouse, and 5 = make own decisions (highest level). The second indicator, the confidence level when making decisions: 1 = very unconfident, 2 = unconfident, 3 = medium, 4 = quite confident, 5 = very confident (highest level). Other elements were similarly coded. The value of each empowerment factor was the average of the component indicators.

The obtained values (based on a 0-5 scale) will be converted to a 0-10 scale. For example, the average resource factor value, 3.2/5, has been converted to 6.4/10.

**The final empowerment index cut-off**

Notably, the study regions were ethnic minority communes, whose EI was significantly lower than that of other regions [3]. Therefore, besides the EI cut-off point of 0.8 that has been used by WEAI to evaluate empowerment (≥ 0.8) ~~and loss of rights (< 0.~~8), in this study, we also applied an additional lower EI cut-off of 0.6 for comparison. Additionally, in the present study, the final EI was simply calculated as the weighted average of 5DE. Based on the cut-off value, the final EI was then rounded up into 0 (disempowered) or 1 (empowered).

**Statistical analysis**

All the statistical analyses were conducted using SPSS 23.0 (SPSS Inc.). The binary regression analysis was performed by assigning the empowerment variable as a dependent variable. The binary logistic model was used to evaluate the factors that impact the final EI. Continuous variables were presented as the mean ± standard deviation. The associations between independent variables with the final empowerment index were determined by one-way analysis of variance (ANOVA) or Student’s t-test, and *p < 0.05* was considered significant difference.

**Results**

**Characteristics of research samples**

The samples were divided almost equally by gender in which women accounted for 48.3% and men accounted for 51.7% of respondents. The average distance from respondents' houses to the nearest urban area was 19.1 km, ~~ranged~~ ranging from 4 to 40 km. The average income per household/month is ~~very low, only~~ 3.8 million VND (about 200 USD/month).~~The average age at first marriage of both sexes combined was 20.2 years old (yo)~~. The average age at first marriage of men (20.7 yo) was higher than that of women (19.3 yo) (*p* = 0.00). Notably, the proportion of child marriage (married under 18 years old) in this area was relatively high, ~~accounted~~ accounting for about 15.8% of the studied populations~~. Especially,~~ the rate of child marriage was more than three times higher in women (23.9%) than in men (7.6%). The average age of all respondents was 36.1 years old, in which the average age of men (38.0 yo) was higher than women (34.6 yo) (p = 0.00). The average number of years attended in school in both sexes combined was 8.7 years ~~and there was a significant difference in this indicator between men and women. Accordingly~~, the average number of years of schooling ~~attended in the school~~ of men (9.6 years) was significantly higher than that of women (7.8 years) (p = 0.00). The average number of children was 2.9. There were no differences in the gender equality awareness index between men and women in this study (p > 0.05) (Table 2).

Table 2. Characteristics of research samples.

| **Research sample** | **Mean** ± **SD** | | |
| --- | --- | --- | --- |
|  | **Total** | **Men** | **Women** |
| **Personal characteristics** | | | |
| **Gender** | 100% | 48.3% | 51.7% |
| **Age** | 36.0 ± 9.2 | 38 ± 8.8*** | 34.5 ± 9.4 |
| **Education** | 8.7 ± 2.8 | 9.4 ± 2.5*** | 7.8 ± 2.8 |
| **The gender equality awareness index** | 3.7 ± 1 | 3.6 ± 1 | 3.7 ± 1.1 |
| **Age at first marriage** | 20 ± 2.9 | 20.7 ± 2.7*** | 19.3 ± 3 |
| **Household characteristics** | | | |
| **Distance from respondents' houses to the nearest urban area** | 19.1 ± 9.7 | NA | NA |
| **Income** | 3.8 ± 1.6 | NA | NA |
| **Number of children** | 2.9 ± 1.2 | NA | NA |
| **Education of children** | 5.3 ± 3.7 | NA | NA |

Statistical analyses were done by Student’s t-test, where ****p* < 0.0001 indicate a significant difference between men and women groups; SD: standard deviation, NA: non-applicable.

**~~Women's empowerment factors in agriculture~~**

**Participation in agricultural production activities**

The level of empowerment in agricultural production activities was ~~has been~~ assessed via two main indicators, namely (i) decision-making power and (ii) confidence levels in decision-making. Noticeably, there was a significant difference between men and women regarding decision-making power in agricultural activities (*p* = 0.00). This rate in food crops farming and livestock raising activities was 40.0% and 33.5%; while in the non-farm economic activities and hired labor reached up to 78.7% and 89.0%, respectively. The proportion of men making their own decisions was also much higher than that of women, especially in the activities of food crop farming (46.2% vs. 11.6%), Cash crop farming (40.7% vs. 0%), livestock raising (64.8% vs. 17.4%), non–farm economic activities (41.4% vs. 5.2%), and hired-labor activities (34.5% vs. 11%) (Fig 1A).

The difference in empowerment between men and women was also clearly observed in ~~the later indicator,~~ the confidence levels in making decisions in agricultural productions (*p* = 0.00). ~~Accordingly,~~ the percentage of women who chose the answer “very unconfident when making decisions” was much higher than that of men (34.2% vs. 0% for food crop farming, 61.9% vs. 29.2% for cash crop farming, 28.4% vs. 0% for livestock raising, 67.1% vs. 53.1% for non-farm activities, and 83.8% vs. 71.0% for hired labor). ~~In parallel,~~ the proportion of men who was “very confident” in decision-making was twice as high of women in all the agricultural activities (81.4% vs. 43.9% for food crop farming, 58.6% vs. 27.7% for cash crop farming, 76.6% vs. 38.7% for livestock raising, 46.9% vs. 11% for non-agricultural activities; 23.4% vs. 11% for hired labor) (Fig 1B).

**Fig 1. The difference between men and women self-assessment in (A) Decision-making power and (B) Confidence level in making decision in agricultural production activities.**

With a cut-off of 0.6, only 21.9% of the women achieved this threshold, compared to 58.4% of the men. None of the women had achieved a threshold of 0.8 compared to 11% of the men (Fig 2A)

**Fig 2. The rate of reach the threshold of empowerment of men and women at cut-off values of 0.6 and 0.8 in (A) Participation in agricultural production activities; (B) Own resources; (C) Control over the use of income; (D) Social participation; (E) Time use; (F) General empowerment in agriculture.**

**Own resources**

Resource ownership was assessed by three indicators, namely (i) property ownership; (ii) to access loans, and (iii) decision-making power in borrowing capital.

For content (i), our results revealed a substantial difference in the property ownership between men and women (p = 0.00), especially in high-value assets. For example, up to 52.4% of men ~~thought that they~~ owned agricultural land ~~was owned~~ by themselves, while, none of the women ~~said that they~~ owned this type of asset (~~0%).~~ For other assets such as cattles, farm equipment, non-agricultural land, houses, and durable goods, the proportion of men who chose the answer of owning it alone (52.4, 64.1, 35.2, 64.1, and 64.2%, respectively) was substantially higher than that of women (6.1, 0.0, 0.0, 6.1, and 6.1%, respectively) (Table 3).

~~For the ability to access loans~~, there was a statistically significant difference between men and women in their ability to access loans (p = 0.03). ~~Accordingly, in the forms of formal and informal credits,~~ the percentage of women respondents answering that they definitely could borrow money was significantly lower than men (formal credit: 34.7% vs. 59.3%; informal credit: 40.8% vs. 55.2%). However, women were more likely than men to access a loan from ~~Only in the form of~~ non-government organizations (NGOs) ~~credit, this rate was higher in women than men~~ (17.7% vs. 11%) but this difference was not significant (p = 0.07) (Table 4).

Regarding decision-making power in borrowing activities (iii), similar to the above two contents, there is also a big difference between men and women. In formal credit institutions, 24.1% of men made ~~said that they~~ ~~make~~ their own decisions when borrowing while none of the women did so. ~~are only 0%~~. This ratio in the form of informal credit is 24.8% for men, compared to 6.1% for women (Table 4). There was no gender difference in decision making for borrowing from NGOs (p > 0.05) (Table 4).

The overall value of the ownership of the resources had also shown the gender difference. Only 40% of the women achieved a cut-off 0.6 compared to 61.3 % of the men. with a cut-off of 0.8, with approximately 85% for both sexes do not achieve this threshold (p > 0.05) (Fig 2B).

**Table 3. Gender differences in owning resources (~~. Data was presented~~ in percentage ~~(%~~**).

| **Answer** | **Agricultural  land** | | **Large  livestock** | | **Farm  equipment** | | **House or  other structures** | | **Large consumer  durables** | | **Land not used  for agricultural purposes** | |
| --- | --- | --- | --- | --- | --- | --- | --- | --- | --- | --- | --- | --- |
|  | **Men** | **Women** | **Men** | **Women** | **Men** | **Women** | **Men** | **Women** | **Men** | **Women** | **Men** | **Women** |
| **No right** | 11.7 | 59.2 | 5.5 | 53.1 | 5.5 | 71.4 | 5.5 | 59.2 | 5.5 | 59.2 | 40.7 | 83.0 |
| **With other non-family member** | 0.0 | 6.1 | 0.0 | 0.0 | 0.0 | 0.0 | 0.0 | 0.0 | 0.0 | 0.0 | 0.0 | 0.0 |
| **With other family member** | 0.0 | 0.0 | 6.2 | 0.0 | 0.0 | 0.0 | 0.0 | 0.0 | 0.0 | 0.0 | 0.0 | 0.0 |
| **With spouse** | 35.9 | 34.1 | 35.9 | 40.8 | 30.3 | 28.6 | 30.3 | 34.7 | 30.3 | 34.7 | 24.1 | 17.0 |
| **Self** | 52.4 | 0.0 | 52.4 | 6.1 | 64.1 | 0.0 | 64.1 | 6.1 | 64.2 | 6.1 | 35.2 | 0.0 |
|  |  |  |  |  |  |  |  |  |  |  |  |  |
|  |  |  |  |  |  |  |  |  |  |  |  |  |

**Show 100% to indicate reading vertically**

Table 4. Gender differences in accessing to loans and lending decision-making power.

| **Ability to take a loan** | | | **Accessing and decisions on loan** | | |
| --- | --- | --- | --- | --- | --- |
| **Answer** | **Men** | **Women** | **Answer** | **Men** | **Women** |
| **NGOs (number (%))** | | | | | |
| **Absolutely impossible** | 111  (76.6%) | 103  (70.1%) | **No right** | 128  (88.3%) | 121  (82.3%) |
| **Impossible** | 0  (0.0%) | 0  (0.0%) | **With other non-family member** | 0  (0.0%) | 0  (0.0%) |
| **Maybe** | 9  (6.2%) | 0  (0.0%) | **With other family member** | 0  (0.0%) | 0  (0.0%) |
| **Possible** | 9  (6.2%) | 18  (12.2%) | **With spouse** | 8  (5.5%) | 17  (11.6%) |
| **Reliably** | 16  (11.0%) | 26  (17.7%) | **Self** | 9  (6.2%) | 9  (6.1%) |
| **Total** | **145**  **(100%)** | **147**  **(100%)** | **Total** | **145**  **(100%)** | **147**  **(100%)** |
| **Formal lender (number (%))** | | | | | |
| **Absolutely impossible** | 59  (40.7%) | 88  (59.9 %) | **No right** | 83  (57.2%)*** | 104  (70.7%) |
| **Impossible** | 0  (0.0%) | 0  (0.0%) | **With other non-family member** | 0  (0.0%) | 0  (0.0%) |
| **Maybe** | 0  (0.0%) | 0  (0.0%) | **With other family member** | 0  (0.0%) | 0  (0.0%) |
| **Possible** | 0  (0.0%) | 8  (5.4%) | **With spouse** | 27  (18.6%)* | 43  (29.3%) |
| **Reliably** | 86  (59.3%)*** | 51  (34.7%) | **Self** | 35  (24.1%)*** | 0  (0.0%) |
| **Total** | **145**  **(100%)** | **147**  **(100%)** | **Total** | **145**  **(100%)** | **147**  **(100%)** |
| **Informal lender and friends or relatives (number (%))** | | | | | |
| **Absolutely impossible** | 48  (33.1%) | 61  (41.5%) | **No right** | 73  (50.3%) | 95  (64.6%) |
| **Impossible** | 0  (0.0%) | 0  (0.0%) | **With other non-family member** | 0  (0.0%) | 0  (0.0%) |
| **Maybe** | 8  (5.5%) | 0  (0.0%) | **With other family member** | 0  (0%) | 0 (0.0%) |
| **Possible** | 9  (6.2%) | 18  (12.2%) | **With spouse** | 36  (24.8%) | 43  (29.3%) |
| **Reliably** | 80  (55.2%)*** | 60  (40.8) | **Self** | 36  (24.8%)*** | 9  (6.1%) |
| **Total** | **145**  **(100%)** | **147**  **(100%)** | **Total** | **145**  **(100%)** | **147**  **(100%)** |

Data was presented as number (%). Statistical analyses were done by Student’s t-test, where **p* < 0.05, ***p* < 0.001 and ****p* < 0.0001 indicate a significant difference between men and womengroups; Missing women: 8.

**Control over the use of income**

There was a statistically significant difference in the family financial control factor between men (8.5/10) and women (5.4/10) (*p* = 0.00). Specifically, the ratio of women who have no decision-making power in the major issues related to family finance usage was up to 61.3% while this ratio in men was only 5.5%. Women were often very unconfident in making big decisions (64.6%) as compared to men (5.5%). Also, the percentage of men (64.1%) who made decisions on their own was much higher than that of women (5.8%). However, the gender difference was much smaller in the proportion of men (29.7%) and women (23.8%) in making small decisions. Remarkably, only in small financial decisions, the proportion of women who responded as “very confident” (40.8%) was higher than men (35.9%) (Table 5).

With a cut-off of 0.6, only 5.5 % of the women achieved this threshold of empowerment in the control over the use of income(?) compared to 60.6% of the men. 5.5% of the women had achieved a threshold of 0.8 compared to 66.5 % of the men *(p = 0.00)* (Fig 2C).

**Table 5. Gender differences on control over the use of income**

| **Decision-making on income generation** | | | | | |
| --- | --- | --- | --- | --- | --- |
| **Answer** | **Men** | **Women** | **Answer** | **Men** | **Women** |
| **Big decisions (number (%))** | | | | | |
| **No right** | 8  (5.5%)*** | 95  (61.3%) | **Veryunconfident** | 8  (5.5%)*** | 95  (64.6%) |
| **With other non-family member** | 0  (0.0%) | 0  (0.0%) | **Unconfident** | 0  (0.0%) | 0  (0.0%) |
| **With other family member** | 0  (0.0%) | 0  (0.0%) | **Medium** | 0  (0.0%)** | 25  (17.0%) |
| **With spouse** | 44  (30.3%) | 51  (32.9%) | **Quite confident** | 0  (0.0%) | 0  (0.0%) |
| **Self** | 93  (64.1%)*** | 9  (5.8%) | **Very confident** | 137 (94.5%)*** | 27  (18.4%) |
| **Total** | **145**  **(100%)** | **155**  **(100%)** | **Total** | **145**  **(100%)** | **147**  **(100%)** |
| **Small decisions (number (%))** | | | | | |
| **No right** | 9  (6.2%)** | 35  (23.8%) | **Very unconfident** | 9  (6.2%)*** | 44  (29.9%) |
| **With other non-family member** | 0  (0.0%) | 0  (0.0%) | **Unconfident** | 0  (0.0%) | 0  (0.0%) |
| **With other family member** | 0  (0.0%) | 0  (0.0%) | **Medium** | 68  (46.9%)** | 43  (29.3%) |
| **With spouse** | 93  (64.1%)* | 77  (52.4%) | **Quite confident** | 16  (11%)* | 0  (0.0%) |
| **Self** | 43  (29.7%) | 35  (23.8%) | **Very confident** | 52  (35.9%) | 60  (40.8%) |
| **Total** | **145**  **(100%)** | **147**  **(100%)** | **Total** | **145**  **(100%)** | **147**  **(100%)** |

Statistical analyses were done by Student’s t-test; **p* < 0.05, ***p* < 0.001 and ****p* < 0.0001 indicate a significant difference between men and women groups; Missing women: 8.

**Social participation**

There are seven types of social participation listed in the questionnaire, namely agricultural organizations, forestry organizations, credit organizations, financial organizations, community support organizations, commercial organizations, charity organizations, and religious organizations. The level of participation of men was higher than women in all the organizations, except for religious organizations. However, these differences were marginal and not statistically significant (*p*> 0.05). There was no statistically significant difference between men and women in the social participation. With a cut-off of 0.6, 21.9% of the women achieved this threshold, compared to 27.9% of the men (p > 0.05). Similarly, at the cut-off of 0.8, there was ~~also~~ no ~~also~~ statistically significant difference. (Fig 2D).

**Time use**

The allocation of working and leisure time was ~~largely~~ different between men and women (*p* = 0.00). Accordingly, 68.4% of women answered that their working time was from night to thirteen hours per day, while only 4.8% of men chose this option. Most of the men in this study (94.5%) had working time from four to eight hours, meaning that women have less rest and leisure time than men. Particularly, 83.2% of women had less than two hours of rest per day while this rate in men was only 5.5%. With a cut-off of 0.6, only 29.4% of the women achieved this threshold, compared to 41.2% of the men. 1.4 % of the women had achieved a threshold of 0.8 compared to 23.7% of the men (p = 0.00) (Fig 2E).

**Assessing the index of empowerment in agriculture**

The general assessment of the above 5 indicators revealed a big difference in the empowerment between men and women (p = 0.00). With a cut-off of 0.6, only 30.3% of the women achieved this threshold, compared to 84.8% of the men. Only 19.4% of the women had achieved a threshold of 0.8 compared to 53.8% of the men (p = 0.00) (Fig 2F).

**Evaluate the factors related to empowerment by the logistic regression model**

To evaluate the empowerment’s related factors, different regression models have been tested. However, the binary logistic regression model has appeared to be the most suitable model for the following reasons: (i) it is logical to use a cut-off to divide the respondents ~~interviewed people~~ into 2 groups (empowered and disempowered ~~loss of rights~~) ~~has been done~~ for all five final empowerment domains and the final EI; (ii) As compared to other regression models, the binary regression can predict variables that can promote the probability of being empowered. This is a crucial factor in recommending specific policies for the ethnic minority areas of Vietnam.

A logistic regression analysis was performed to assess the factors associated with empowerment ~~for the dependent variable~~ at both 0.6 and 0.8 cut-off values. Because the percentage of both men and women not reaching the empowerment threshold of 0.8 is very high, the regression results at this value would not be very meaningful. Therefore, we only focused on the regression results at the cut-off of 0.6. The results of the general conformity test showed *p* = 0.000, indicating that the model can explain well ~~for~~ the hypotheses with the given independent variables. The -2 log-likelihood value was used ~~qualified~~ to evaluate the suitability of the model in explaining the hypotheses. The correct prediction rate of the model was 72.3%. Among eight independent factors that were contemporaneously included in the model to evaluate their impact on the dependent variable (probability of reaching the empowered threshold), five factors were statistically significant (*p*< 0.05), namely (i) the educational level (measured by the number of years of schooling), (ii) the age at first marriage, (iii) the distance to the nearest urban area, (iv) the children’s highest years of schooling, and (v) the number of children, in which (i), (ii), and (iv) showed a positive correlation while (iii) and (v) showed a negative correlation to the probability of empowerment (Fig 2F). Particularly, the ~~higher~~ number of years of schooling, age at first marriage, and the number of children’s years of schooling, the greater chance of reaching the empowered threshold. If each of these factors increased by 1 unit (1 year) while all the other factors remained the same, probability of reaching the empowered threshold increased by 1.22, 1.14, and 1.17 times, respectively. On the other hand, the larger the distance to the nearest urban and the number of children in the family, the lower the probability of reaching the empowered threshold ~~one may be granted~~. If each of these factors increased by 1 unit (1 km for the distance and 1 child for the number of children), the probability of being empowered decreased by 0.96 and 0.65 times, respectively (Table 6).

**Table 6. Regression results of factors ~~that~~ related the probability of empowerment.**

| **Factors** | | **Variables in the equation** | | | | | |
| --- | --- | --- | --- | --- | --- | --- | --- |
|  |  | **B** | **S.E.** | **Wald** | **Df** | ***p* value** | **Exp(B)** |
|  | **Age** | -.033 | .031 | 1.146 | 1 | .284 | **.968** |
|  | **Education** | .199 | .074 | 7.253 | 1 | **.007** | **1.220** |
|  | **Age at first marriage** | .134 | .058 | 5.451 | 1 | **.020** | **1.144** |
|  | **Distance to the nearest urban** | -.040 | .015 | 6.683 | 1 | **.010** | **.961** |
|  | **Children’s education** | .159 | .079 | 4.072 | 1 | **.044** | **1.172** |
|  | **Income** | -.150 | .337 | .197 | 1 | .657 | **.861** |
|  | **Gender equality awareness** | -.221 | .192 | 1.327 | 1 | .249 | **.802** |
|  | **Number of children** | -.428 | .170 | 6.314 | 1 | **.012** | **.652** |
|  | **Constant** | -.592 | 1.621 | .133 | 1 | .715 | **.553** |

B:Estimated coefficient; S.E: standard error of the mean; Wald: wald test meaning; Df:the degrees of freedom coefficients; *p* value: the level of statistical significance; Exp(B): estimated coefficient weights; number of observations: 155.

**Discussion**

In general, our results reflected the reality of gender differences in empowerment in agriculture-related activities in the survey areas. Particularly, the assessment of the related factors of empowerment would be an important basis for proposing policies that seek to boost the level of women's empowerment in agriculture. Similar to the previous studies in Vietnam [26], our results also showed the absolute control of men on agricultural production-related decisions. One of the reasons for this was that the rural society of Vietnam is still being influenced by Confucius' views, in which men had higher positions and full power to make decisions in the family, whereas, women were obligated to perform household chores ~~serve~~ [10,16,27].

In addition, our results also indicated that most of the women in the rural area have less rights than men of property ownership, especially for valuable properties such as farm equipment and non-agricultural land. This point had also been raised by most of the previous studies in Vietnam and some other developing countries [28,29]. This finding could be partly explained by "culture" that was considered as one of the intangible barriers prohibiting women's property ownership. More specifically, gender stereotyping s~~tereotypes~~ has prevented (or restricted) ~~eliminated~~ women from property ownership claims [30–33].

In concordance with most empowerment studies in developing countries ~~have has commonly been reported often found women to have less power than men in credit-related decisions [34,35]. In concordance with that, our~~ the present study also showed women have much less power than men in credit-related decisions. This observation can be explained by two main reasons: (i) in developing countries, the majority of the household heads ~~householders~~ were men, who were legally recognized and had the right to own valuable assets. Therefore, they also have the ~~top~~ authority to mortgage these assets for loans; (ii) cultural, legal, and economic barriers, the level of financial awareness, or differences in gender behaviour also hindered women's decisions on this issue. Our results also show that men are still the main decision makers in household expenditure which is different from most studies showing that women and men make joint decisions on household expenditures [10,11].This observation may be due to the fact that (i) the main income earners in these households are mostly men; thus the ownership and spending decisions also belong to them; (ii) the women in the central of Vietnam, ~~the research area~~, are known for their endurance and undemanding behaviour ~~that has led to limitations in their bargaining and spending decision-making ability.~~

Among all the investigated indicators in this study, social participation is the only indicator that did not show any gender differences. However, the level of social participation of both men and women in the research area was very low. The possible reasons for such low participation in social activities are as follows: ~~Possibly,~~ (i) the closed living space, small-scale farming practices, and self-sufficiency have created a feeling of reluctance to expand the relationships and to participate in socio-political activities; and (ii) the income of the majority of households in the study area is still very low, so they must spend more time on labor to generate income for the family.

The time use is an indicator that was clearly different between men and women. This result is also confirmed by most of the previous studies on the same topic in the rural areas of Vietnam in particular and the rural areas of other Asian countries in general [36,37]. Women in the research areas, apart from participating in farming, also must undertake all the housework and taking care of the family members (~~in unpaid work~~). As mentioned above, due to gender role stereotyping, few men were engaged in ~~volunteered to do~~ housework with their wives. They often shun the housework and considered it as a women's duty. This is also the main reason why women do not have much rest and leisure time ~~to recreate labor power~~.

Taken together, our results showed that women have much lesser ~~lower~~ rights than men in all 5 indicators. The final EI also showed that nearly 70% of women in this study did not reach the empowered threshold in agriculture. This finding is contradictory to several recent studies in other Southeast Asia regions, which indicated that women have higher rights than men in production-related activities and control the family’s income [10]. While the education level, the distance to the nearest urban area, and the number of children is related to women's empowerment were the common findings in most of the empowerment studies [18,20–22,24,34], the age at first marriage, the highest education of children detected in this study were novel factors. In particular, the age of first marriage is closely related to empowerment. Noticeably, child marriages were still common in this study area, which accounted for 15.8% of the total sample. This fact has been one of the main reasons why women in this study had lower voices and power in decision-making and were dependent on their husband and husband's family [38,39].Child-marriages were the major factor that was directly affecting the capacity of women's empowerment in general and rural women in particular. Lastly, the highest education level of the children also had an impact on the empowerment of women. It was believed that the daily interactions in the family has changed the perception of family members, especially the right of a mother to participate and decide different family issues. These ~~cognitive~~ changes will encourage individuals to change gender practice, increasing the likelihood of empowerment. The ~~remained other~~ factors such as age, income, and awareness of gender equality were ~~had no~~ related on the level of empowerment.

**Conclusion and policy recommendations**

Firstly, in the present study, we found that in all five assessed factors women played a lesser role than the men in rural agricultural setting ~~were~~, across socio-demographic variables. Resource ownership, spend time, and household financial control were are typical elements.

Secondly, 70% of women in this study did not reach the empowered threshold in agriculture signified a large gender gap in these specific geographic areas, and suggested an urgent need for specific and timely policy interventions to increase women's empowerment in these regions.

Thirdly, ~~the final~~ logistic regression results strongly suggested that, in order to reduce ~~narrow down~~ the gender gap in empowerment, policies need to be focused on the following issues:

(i) improving the educational level for people in the remote ~~distant~~ areas, especially young women and girls ~~and the coming generations~~. The relationship of educational attainment and children's education on the probability of empowerment indicates the need for policy interventions in this issue;

(ii) increasing the age at first marriage. Our results revealed that child marriage in mountainous regions of Vietnam is still common ~~ly existed.~~ Marriage at the age of under 18 plus high fertility bring multiple health implications and reduce ~~minimize~~ the opportunity to access basic social services. These are the obvious barriers that hinder the empowerment of rural women today. Low education - early marriage – giving birth to too many children, abd poverty appears to be a "vicious circle." To be able to escape from this vicious ~~non-end~~ circle, it is requires a "kick from outside" [40], of which livelihood support and communication for behavior change must be the key policies and done simultaneously;

(iii) promoting urbanization. The closer to the urban area, the higher probability of empowerment will be, indicating an obvious influence of the urban on the surrounding rural areas. However, urbanization is a lengthy process and its outcomes depend on multiple factors. For mountainous regions, in particular, this process would be even more difficult. The typical characteristics of the city such as diverse forms of communication, the development of culture, were specific policy implications that can be applied in raising the awareness of rural people about the rights of themselves and the community in agriculture.

Fourthly, Our measurement method (~~inheriting~~ using variables and calculating weights of WEAI) is completely quantitative, while empowerment is a more qualitative concept. In the process of collecting data for this study, we realized that for many issues, the collection of qualitative data through individual in-depth interviews or focus group discussions could exploit more specific information and thus provide higher quality data. we are trying to combine qualitative and quantitative research methods in subsequent studies on empowerment in ethnic minority areas, central Vietnam.

Limitations of this study

**Acknowledgments**

The authors would like to thank Dr. Vinay Bharadwaj Tatipamula and Dr. Ha Thi Nguyen (Institute of Research and Development, Duy Tan University, Danang, Vietnam) for the critical reading of the manuscript.

**Conflict of interest statement**

The other authors report no conflicts of interest.

**References**

1. Kabeer N. Resources, agency, achievements: Reflections on the measurement of women’s empowerment. Dev Change. 1999;30:435–464. doi:10.1111/1467-7660.00125
2. World Bank. Empowerment in practice: Analysis and implementation. 2007. Available: http://siteresources.worldbank.org/WBI/Resources/EmpowermentLearningModulebody.pdf
3. Alkire S, Meinzen-Dick R, Peterman A, Quisumbing A, Seymour G, Vaz A. The women’s empowerment in agriculture index. World Dev. 2013;52:71–91. doi:10.1016/j.worlddev.2013.06.007
4. CGIAR. CGIAR Annual report 2014: Featuring nutrition and health. 2014. Available: https://library.cgiar.org/handle/10947/4088
5. Belay F, Oljira A. Gender role in agricultural activities in ethiopia: Country review. J Cult Soc Dev. 2016;22:1–7.
6. FAO. Women in agriculture closing the gender gap for development. 2011. Available: https://www.fao.org/publications/sofa/2010-11/en/
7. FAO. The state of food and agriculture. Population and development review. 2017. Available: https://www.fao.org/3/I7658EN/i7658en.pdf
8. Manfre C, Rubin D, Allen A, Summerfield G. Reducing the gender gap in agricultural extension and advisory services: How to find the best fit for men and women farmers. Meas Brief. 2013;2:1-10.
9. Harper S, Zeller D, Hauzer M, Pauly D, Sumaila UR. Women and fisheries: Contribution to food security and local economies. Mar Policy. 2013;39:56–63. doi:10.1016/j.marpol.2012.10.018
10. Akter S, Rutsaert P. Women’s empowerment and gender equity in agriculture: A different perspective from Southeast Asia. Food Policy. 2017;69:270–279. doi:10.1016/j.foodpol.2017.05.003
11. Alkire S. Subjective quantitative studies of human agency. Soc Indic Res. 2005;74:217–260. doi:10.1007/s11205-005-6525-0
12. Mason KO, Smith HL. Women’s empowerment and social context: Results from five Asian countries. Gender and Development Group, World Bank, Washington, DC. 2003;53:1-28.
13. Kishor S, Subaiya L. Understanding women’s empowerment: A comparative analysis of demographic and health surveys (DHS) data. DHS Comp Reports No 20. 2008. Available: https://dhsprogram.com/pubs/pdf/CR20/CR20.pdf
14. Fletschner D, Kenney L. Rural women’s access to financial services: Credit, savings, and insurance. Agric Dev Econ Div. 2011;1–30. doi:10.1007/978-94-017-8616-4_8
15. Akter S, Krupnik TJ, Rossi F, Khanam F. The influence of gender and product design on farmers’ preferences for weather-indexed crop insurance. Glob Environ Chang. 2016;38:217–229. doi:10.1016/j.gloenvcha.2016.03.010
16. Maligalig R, Demont M, Umberger WJ, Peralta A. Off-farm employment increases women’s empowerment: Evidence from rice farms in the Philippines. J Rural Stud. 2019;71:62–72. doi:10.1016/j.jrurstud.2019.09.002
17. Allendorf K. Do women’s land rights promote empowerment and child health in Nepal? World Dev. 2007;35:1975–1988. doi:10.1016/j.worlddev.2006.12.005
18. Allendorf K. Women’s agency and the quality of family relationships in India. Popul Res Policy Rev. 2012;31:187–206. doi:10.1007/s11113-012-9228-7
19. Gupta K, Yesudian PP. Evidence of women’s empowerment in India: A study of socio-spatial disparities. GeoJournal. 2006;65:365–380. doi:10.1007/s10708-006-7556-z
20. Hindin MJ. Women’s power and anthropometric status in Zimbabwe. Soc Sci Med. 2000;51:1517–1528. doi:10.1016/S0277-9536(00)00051-4
21. Jejeebhoy SJ, Sathar ZA. Women’s autonomy in India and Pakistan: The influence of religion and region. Popul Dev Rev. 2001;27:687–712. doi:10.1111/j.1728-4457.2001.00687.x
22. Malhotra A, Mather M. Do schooling and work empower women in developing countries? Gender and domestic decisions in Sri Lanka. Sociol Forum. 1997;12:599–630. doi:10.1023/A:1022126824127
23. Ragsdale K, Read-Wahidi MR, Wei T, Martey E, Goldsmith P. Using the WEAI+ to explore gender equity and agricultural empowerment: Baseline evidence among men and women smallholder farmers in Ghana’s Northern Region. J Rural Stud. 2018;64:123–134. doi:10.1016/j.jrurstud.2018.09.013
24. Roy BTK, Niranjan S. Indicators of women’s empowerment in India. Asia Pac Popul J. 2004;19:23–38. doi:10.18356/b8bd2326-en
25. United Nations Viet Nam. The review report on the implementation of the national strategy on gender equality (NSGE) 2011-2020. Available: https://vietnam.un.org/en/139279-review-report-implementation-national-strategy-gender-equality-nsge-2011-2020
26. Teerawichitchainan B, Knodel J, Loi VM, Huy VT. The gender division of household labor in vietnam: Cohort trends and regional variations. J Comp Fam Stud. 2010;41:57–86. doi:10.3138/jcfs.41.1.57
27. Nguyen TH. The Vietnamese concept of a feminine ideal and the images of Australian women in Olga Masters ’ stories. Gender Forum: An internet platform for gender and women’s studies. 2013;45:1–10.
28. Kieran C, Sproule K, Doss C, Quisumbing A, Kim SM. Examining gender inequalities in land rights indicators in Asia. IFPRI Discuss Pap. 2015;46:119–138. doi:10.1111/agec.12202
29. Beuchelta IFT, et al. Social networks as means of information exchange and risk-management - a case study from Northern Viet Nam. Conference on International Agricultural Research for Development. 2005:1–3. Available: http://www.asareca.org/paap/uploads/publications/Deutscher Tropentag paper presented 2005.pdf
30. Bayisenge J. Women’s experiences of the land tenure reform programme in Rwanda. Int J Gend Women’s Stud. 2015;3:118–133. doi:10.15640/ijgws.v3n1p12
31. Chigbu UE. Repositioning culture for development: Women and development in a Nigerian rural community. Community, Work Fam. 2014;18:334–350. doi:10.1080/13668803.2014.981506
32. Chigbu UE. Masculinity, men and patriarchal issues aside: How do women’s actions impede women’s access to land? Matters arising from a peri-rural community in Nigeria. Land Use Policy. 2019;81:39–48. doi:10.1016/j.landusepol.2018.10.033
33. Lambrecht I, Vanlauwe B, Maertens M. Agricultural extension in Eastern Democratic Republic of Congo: Does gender matter? Eur Rev Agric Econ. 2016;43:841–874. doi:10.1093/erae/jbv039
34. Fletschner D. Rural women’s access to credit: Market imperfections and intrahousehold dynamics. World Dev. 2009;37:618–631. doi:10.1016/j.worlddev.2008.08.005
35. Lusardi A, Schneider D, Tufano P. Financially fragile households: Evidence and implications. Brookings Pap Econ Act. 2011;83–134. doi:10.1353/eca.2011.0002
36. Cook S, Dong XY. Harsh Choices: Chinese women’s paid work and unpaid care responsibilities under economic reform. Dev Change. 2011;42:947–965. doi:10.1111/j.1467-7660.2011.01721.x
37. de Bruin A, Liu N. The urbanization-household gender inequality nexus: Evidence from time allocation in China. China Econ Rev. 2020;101301. doi:10.1016/j.chieco.2019.05.001
38. Jain S, Kurz K. New insights on preventing child marriage: A global analysis of factors and programs. Int Cent Res Women. 2007;1–53.
39. K.G.Santhya, Usha Ram R, Acharya SJ. Associations between early marriage and young women’s marital and reproductive health outcomes: Evidence from India. Int Perspect Sex Reprod Health. 2010;36:132–139. doi:10.1363/3613210
40. Siti Rohima SR et al. Vicious circle analysis of poverty and entrepreneurship. IOSR J Bus Manag. 2013;7:33–46. doi:10.9790/487x-0713346
